# Polymer fibrous aerogels for thermal insulation: from molecular building blocks to structural reliability

**DOI:** 10.1039/d6ra08043j

**Published:** 2026-10-02

**Authors:** Xuepeng Ni, Jingyu Guan, Jie Dou, Jiaming Guo, Zhe Cui, Xiaojuan Wang, Caixia Ren, Liyin Hou, Qi Wang

**Affiliations:** a Shandong Engineering Research Center of Low-Carbon Energy Internet of Things Technology, School of Energy and Constructional Engineering, Shandong Huayu University of Technology Dezhou 253034 P.R. China hly@huayu.edu.cn; b Christopher Ingold Laboratory, Department of Chemistry, University College London London WC1H 0AJ UK zhe.cui@ucl.ac.uk; c School of Environment and Safety Engineering, Nanjing Polytechnic Institute Nanjing 210048 China sdzzwangqi@163.com; d China Nuclear Power Engineering Co., Ltd Beijing 100840 China

## Abstract

Polymer fibrous aerogels combine the low density and high porosity of conventional aerogels with the continuity and deformability of fibrous networks, offering a route to lightweight thermal protection that can remain mechanically compliant. Their design is intrinsically coupled: reducing solid content, refining pores, strengthening junctions, or introducing functional phases can suppress one heat-transfer pathway while intensifying another or destabilizing the network. This review adopts retained thermal insulation after service exposure, rather than the minimum conductivity of the pristine specimen, as its central evaluation criterion. We trace how polymer chemistry, fiber formation, three-dimensional assembly, interfiber stabilization, and drying govern solid and gas conduction, thermal radiation, residual convection, and moisture-mediated transport, and how these pathways evolve after deformation, humidity, heating, and fire exposure. Cellulose-based, other bio-derived, aramid, PBO, polyimide, polyamide-imide, thermoplastic, and char-forming systems are compared using measurable structural descriptors and matched test conditions rather than composition labels alone. Across these material classes, durable insulation emerges within a structural window of sparse load-bearing fibers, localized mechanically effective junctions, controlled pore connectivity, and spatially confined functional phases. The resulting processing–structure–transport–retention framework identifies the evidence required to translate polymer fibrous aerogels from record-setting laboratory specimens into reproducible insulation systems with verifiable service reliability.

## Abstract 2

Polymer fibrous aerogels combine the low density and high porosity of conventional aerogels with the continuity and deformability of fibrous networks, offering a route to lightweight thermal protection that can remain mechanically compliant.

## Graphical abstract

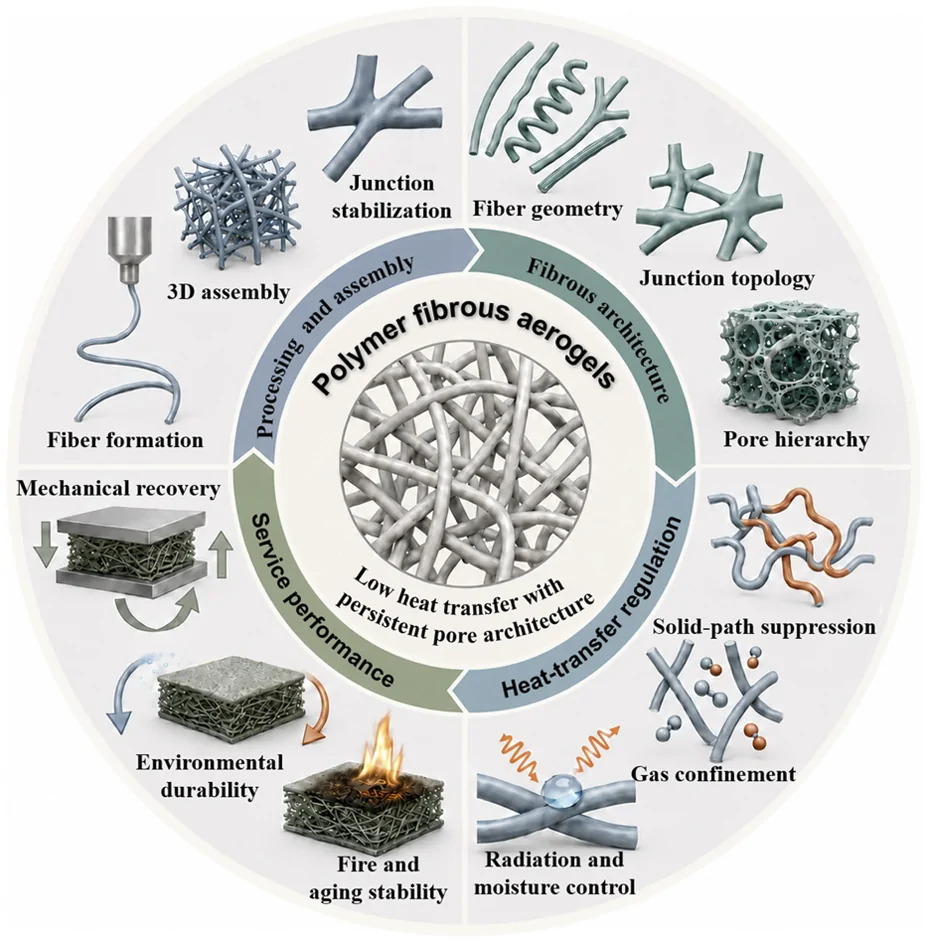

## 1 Introduction

Thermal insulation is central to energy conservation and temperature control in buildings, transportation, protective clothing, electronics, and aerospace systems. In these settings, low heat transfer must be maintained under simultaneous constraints of mass, thickness, deformation, humidity, thermal cycling, and fire exposure. Conventional polymer foams are lightweight and scalable but remain vulnerable to flammability, creep, cell coarsening, and restricted service temperatures. Mineral-fiber mats tolerate deformation but generally require greater thickness and may permit air movement or fiber release. Silica and ceramic aerogels suppress gas conduction effectively, yet monoliths are brittle, whereas reinforced blankets may undergo dusting, shrinkage, interfacial failure, or reinforcement-induced thermal bridging. Reviews of hybrid aerogels,^1^ biomass-derived aerogels,^2^ nanocellulose aerogels,^3^ and aerogel fibers^4^ point to a common materials challenge: the solid fraction must be minimized to restrict heat flow but remain sufficiently connected to preserve the pore architecture.

Polymer fibrous aerogels address this conflict through continuous fiber networks that simultaneously define the pore space and carry mechanical load. Interconnected microfibers, ultrafine fibers, nanofibers, or fibrils can form mechanically percolated skeletons at low solid contents while introducing tortuous heat-transfer pathways, multiscale pores, and abundant interfacial resistance. Related fibrous aerogel systems provide valuable mechanistic benchmarks. Fiber-reinforced silica aerogels reveal the effects of reinforcement and solid bridging,^5^ while inorganic dual networks demonstrate the stabilizing role of secondary skeletons.^6^ Scalable aluminosilicate-fiber composites^7^ and lamellar ceramic nanofibrous aerogels^8^ provide high-temperature references. Multifunctional aerogels,^9^ radiative-transfer analyses,^10^ hygrothermal studies,^11^ and mixed-fiber composites^12^ further clarify the influences of functional phases, radiation, environmental conditioning, and reinforcement continuity.

The field has consequently moved from producing fragile, ultralight specimens toward designing persistent and manufacturable fibrous architectures. Zhang *et al.* used hierarchical interface engineering to strengthen nanocellulosic aerogels,^13^ whereas Zheng *et al.* localized covalent fusion at mechanically important contacts to obtain robust aerogels for deployable thermal protection.^14^ In aramid systems, Hu *et al.* regulated proton donation during network regeneration,^15^ Wu *et al.* used acid-assisted contact consolidation,^16^ and Li *et al.* introduced branched fibrils to combine low heat transfer with compressive strength.^17^ For polyimide (PI), Wang *et al.* encoded elasticity through curly nanofibers,^18^ while Li *et al.* constructed spatial gradients for cryogenic and high-temperature environments.^19^ Manufacturing studies by Yu *et al.* on sea-island melt-spun ultrafine fibers,^20^ Li *et al.* on large-scale isotropic assembly,^21^ Xue *et al.* on direct ink writing,^22^ Zhu *et al.* on ambient drying,^23^ and Li *et al.* on recyclable PI processing^24^ further demonstrate that thermal performance depends on how fiber geometry, dispersion, junction topology, pore hierarchy, and drying evolve together.

Despite these advances, the field still lacks a consistent causal connection between processing, fibrous architecture, heat-transfer pathways, and retained service performance. Effective conductivity contains coupled contributions from the solid skeleton, confined gas, radiation, residual convection, and sorbed or condensed moisture. Each contribution changes during drying, compression, heating, and aging. Hygrothermal measurements reveal that humidity and temperature can alter both the pore gas and the network itself;^11^ Fang *et al.* further showed through structural modeling that fiber orientation and pore geometry cannot be separated from heat flow.^25^ Thermal treatment can reconstruct or densify reinforced networks,^26^ and comparative measurements confirm the strong dependence of conductivity on density, reinforcement state, and test conditions.^27^ Structural regulation is nonmonotonic: additional junctions preserve thickness but enlarge conductive contacts; finer fibers reduce the required solid fraction but may aggregate; and opacifiers suppress radiation while creating new solid paths. Minimum room-temperature conductivity alone is consequently an inadequate measure of material quality.

This review addresses these limitations through a processing–structure–transport–retention framework (Fig. 1). Section 2 establishes how polymer chemistry defines the accessible fiber, junction, and service windows. Section 3 follows the transformation from fibers or fibrils to a dried three-dimensional network. Section 4 links fiber connectivity, pore topology, gradients, hybrid phases, and environmental exposure to the individual modes of heat transfer. Section 5 then evaluates whether these structural benefits survive the boundary conditions of clothing, buildings, transportation, electronics, and extreme environments. Rather than compiling isolated record values, the review compares representative studies, identifies overdesign-induced failure modes, and develops application-specific criteria for structural reliability and scale-up.

**Fig. 1:**
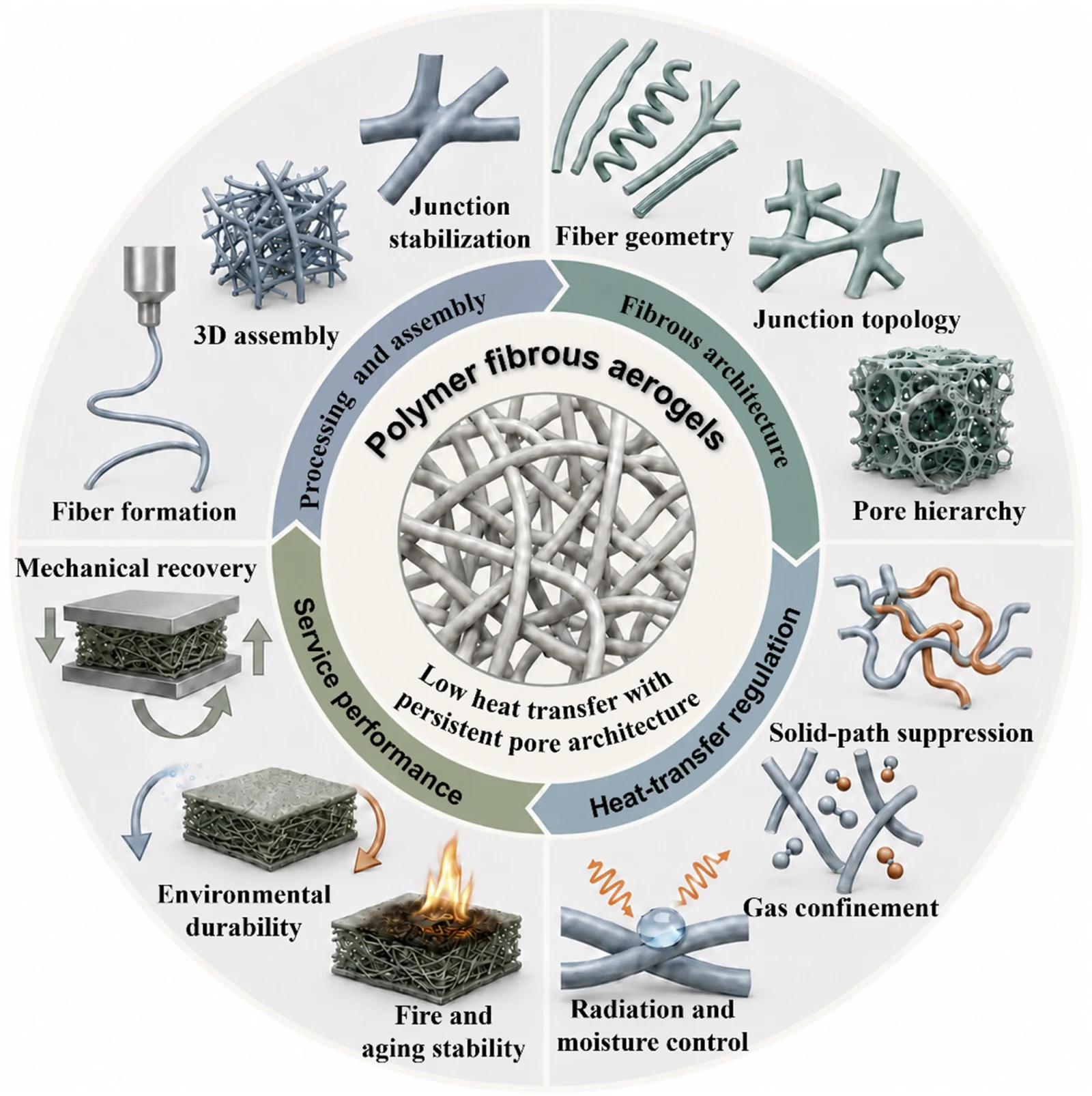
Design framework for polymer fibrous aerogels with low heat transfer and persistent pore architectures.

## 2 Polymer building blocks and design principles

### 2.1 Molecular design principles for fibrous aerogels

Molecular design in fibrous aerogels should be considered in relation to network formation rather than polymer selection alone. The polymer must first be converted into fibers or fibrils with sufficient aspect ratio, then dispersed or deposited without severe aggregation, and finally stabilized at fiber contacts without filling the surrounding pore space. Its molecular structure influences thermal insulation indirectly by controlling three successive processes: fiber formation, junction construction, and structural retention during drying and service. Studies on aramid nanofibers, cellulose nanofibrils, polyimide networks, and thermoplastic microfibers have shown that polymers with similar intrinsic thermal stability can produce markedly different aerogel structures because of differences in their fibrillation behavior, surface chemistry, and interfiber interactions.^15–20,28–32^

The polymer backbone primarily determines whether the fibers can withstand processing and subsequent thermal exposure. Rigid aromatic or heterocyclic chains, as found in aramids and polyimides, restrict segmental motion and generally provide high modulus, low creep, and resistance to thermal shrinkage.^15–18,31–41^ These characteristics are advantageous for preserving the pore network at elevated temperatures. However, the same strong intermolecular interactions that provide structural stability may also make the polymer difficult to dissolve, fibrillate, or redisperse. Aramid fibers, for example, must commonly be converted into nanoscale fibrils through controlled deprotonation or related disassembly processes before a homogeneous three-dimensional network can be formed.^42,43^ Polyimide systems often avoid direct processing of the fully imidized polymer by constructing the porous network from soluble poly(amic acid) precursors, followed by imidization and crosslinking.^18,31–41,44^ Backbone stability must be considered together with the chemical route by which that backbone can be processed into a fibrous network.

Functional groups on the fiber surface subsequently govern dispersion and junction formation. Hydroxyl, amide, carboxyl, and ionic groups promote solvent compatibility and generate hydrogen-bonded, ionic, or covalent contacts between adjacent fibers.^13,14,29,30,45–65^ Cellulose nanofibrils illustrate this advantage: their abundant hydroxyl groups enable aqueous dispersion and extensive hydrogen bonding, allowing lightweight networks to be assembled without a dense polymer binder.^13,29,30,45,47^ Nevertheless, these polar groups also increase moisture uptake. Sorbed water can plasticize the network, alter fiber contacts, and increase thermal conductivity because water conducts heat more effectively than air.^11,55–58,63–65^ Surface modification or covalent crosslinking can improve moisture resistance, but excessive reaction may coat the fibers, enlarge the junctions, or produce continuous polymer-rich domains. Such densification shortens the solid heat-transfer path and can offset the benefit gained from improved mechanical stability.^14,55,66–68^ The objective is to stabilize discrete fiber contacts while retaining open pores and a low solid fraction.

The appropriate molecular design also depends on the assembly route. For preformed nanofibers, surface charge, solvent affinity, and reversible interfiber interactions are particularly important because they determine dispersion and aggregation during gelation and freezing.^13–17,21,28–30,42,45,69^ For electrospun or melt-spun fibers, molecular weight, chain entanglement, crystallization, and melt or solution rheology govern fiber continuity and diameter, whereas subsequent welding or crosslinking determines whether the deposited network survives drying and compression.^18,20,31–41,70–73^*In situ* polymerizable systems offer greater control over covalent bonding, but the reaction must remain localized at fiber contacts; uncontrolled polymer growth within the pores increases density and solid conduction.^14,40,41,67,68,74–78^ Molecular requirements should be defined for the intended fabrication route rather than treated as universal material-selection criteria.

Accordingly, a suitable polymer for a fibrous aerogel should satisfy four connected requirements: it must be processable into high-aspect-ratio fibers, provide controllable interactions at fiber contacts, resist thermal and environmental relaxation, and preserve a low-volume-fraction heat-transfer network. These requirements rarely coincide in a single molecular feature. Rigid backbones improve thermal stability but complicate processing; polar groups assist assembly but promote moisture sorption; and crosslinkable groups strengthen junctions but may densify the network. Effective molecular design depends on balancing these competing effects and, where necessary, separating their functions through copolymerization, surface modification, blending, or localized crosslinking. The best precursor is not simply the polymer with the highest decomposition temperature, but the one whose molecular architecture can be reproducibly translated into a sparse, well-connected fibrous network that remains stable under the intended thermal, mechanical, and humid conditions.

### 2.2 Cellulose-based fibrous systems

Cellulose is the most extensively investigated renewable precursor for polymer fibrous aerogels because it is available as cellulose nanofibrils, bacterial-cellulose ribbons, regenerated microfilaments, pulp fibers, and short natural fibers. These forms perform different structural functions. Nanofibrils generate numerous contact points and form continuous skeletons at low solid contents, whereas microfilaments and short fibers span larger pores and distribute stress over longer distances. Their hydroxyl-rich surfaces also enable hydrogen bonding, ionic coordination, and covalent coupling, making interfiber junctions more readily adjustable than those of chemically inert thermoplastic fibers. Zhang *et al.* used hierarchical interface engineering to strengthen the contacts between cellulose nanofibers and silane-derived components.^13^ The resulting aerogel combined a thermal conductivity of approximately 35 mW m^−1^ K^−1^ with improved compression resistance, showing that mechanical reinforcement can be achieved without uniformly densifying the pore walls. Zheng *et al.* adopted a more localized strategy by introducing covalent fusion specifically at mechanically important bacterial-cellulose contacts.^14^ The resulting aerogel retained a density of 16.1 mg cm^−3^, a porosity of 98.8%, and a thermal conductivity of 27.0 ± 0.2 mW m^−1^ K^−1^, while surviving 10 000 compression cycles, 1000 folding cycles, and 10 000 shear cycles without pronounced structural deterioration. Mechanical reliability is governed primarily by the strength and spatial distribution of critical fiber junctions rather than by a general increase in solid content.^30,63–65^

This principle remains applicable when cellulose is recovered from cotton waste, wastepaper, or other recycled sources. Wei *et al.* induced partial aggregation of waste-cotton cellulose into slender microfilaments rather than fully individualizing it into isolated nanofibrils.^45^ These continuous load-bearing paths yielded a thermal conductivity of 21.5 ± 0.2 mW m^−1^ K^−1^ and a height-recovery ratio of 91.26% after 100 compression cycles. Controlled aggregation can improve fatigue resistance without markedly increasing heat transfer, provided that the aggregates remain slender and interconnected instead of developing into compact bundles. Su *et al.* similarly converted cotton waste into reinforced cellulose composite aerogels, demonstrating that recovered fibers can function as structural elements rather than merely serving as low-cost replacements for virgin cellulose.^46^ Ionic association provides another way to stabilize the network. In an alginate–cellulose nanofiber aerogel, Ca^2+^ crosslinking produced an anisotropic structure with a density of 0.015 g cm^−3^ and a thermal conductivity of 31.5 mW m^−1^ K^−1^.^47^ The aerogel also exhibited a burning velocity of only 0.053 mm s^−1^ and self-extinguishing behavior. These properties arose from the combination of cellulose-nanofiber reinforcement with the ionic crosslinkability and fire response of alginate.

The amount of reinforcing fiber must nevertheless remain within an appropriate range. Wu *et al.* introduced cellulose fibers recovered from office wastepaper into konjac-glucomannan/starch aerogels.^48^ The resulting materials had densities of 30.1–44.9 mg cm^−3^ and porosities of 95.1–97.0%. Increasing the wastepaper-fiber content from 0.1 to 0.5 wt% refined the pores, increased the tortuosity of the heat-transfer path, reduced hygroscopicity, and lowered the thermal conductivity to 33.5 mW m^−1^ K^−1^. Raising the fiber content further to 1.5 wt% reversed this improvement because the additional cellulose increased solid conduction and compacted the pore walls. The contribution of renewable fibers is nonmonotonic: they improve insulation only while pore stabilization and subdivision outweigh the additional solid heat-transfer pathways introduced into the network.

Agricultural residues extend the accessible range of fiber dimensions and native geometries. Yi *et al.* retained the microscale morphology of cellulose fibers extracted from cornstalk rind and combined them with carboxymethyl cellulose (CMC) and PVA through freeze–thaw treatment followed by freeze-drying.^49^ Hydrogen bonding among the cornstalk fibers, CMC, and PVA generated an interconnected network with a density of 0.0198 g cm^−3^ and a porosity of approximately 95%. After hydrophobic modification, the aerogel exhibited a water-contact angle of 159°, a compressive strength of approximately 200 kPa, and a thermal conductivity of approximately 53 mW m^−1^ K^−1^. As shown in Fig. 2a–c, the cornstalk fibers form the principal load-bearing framework, while CMC and PVA bridge adjacent fibers through multiple hydrogen-bonding interactions. The cross-sectional morphology confirms that the micrometer-scale fibers remain embedded within an open porous matrix, and the stress–strain curves show that retention of the cellulose skeleton increases resistance to large compressive deformation.

**Fig. 2:**
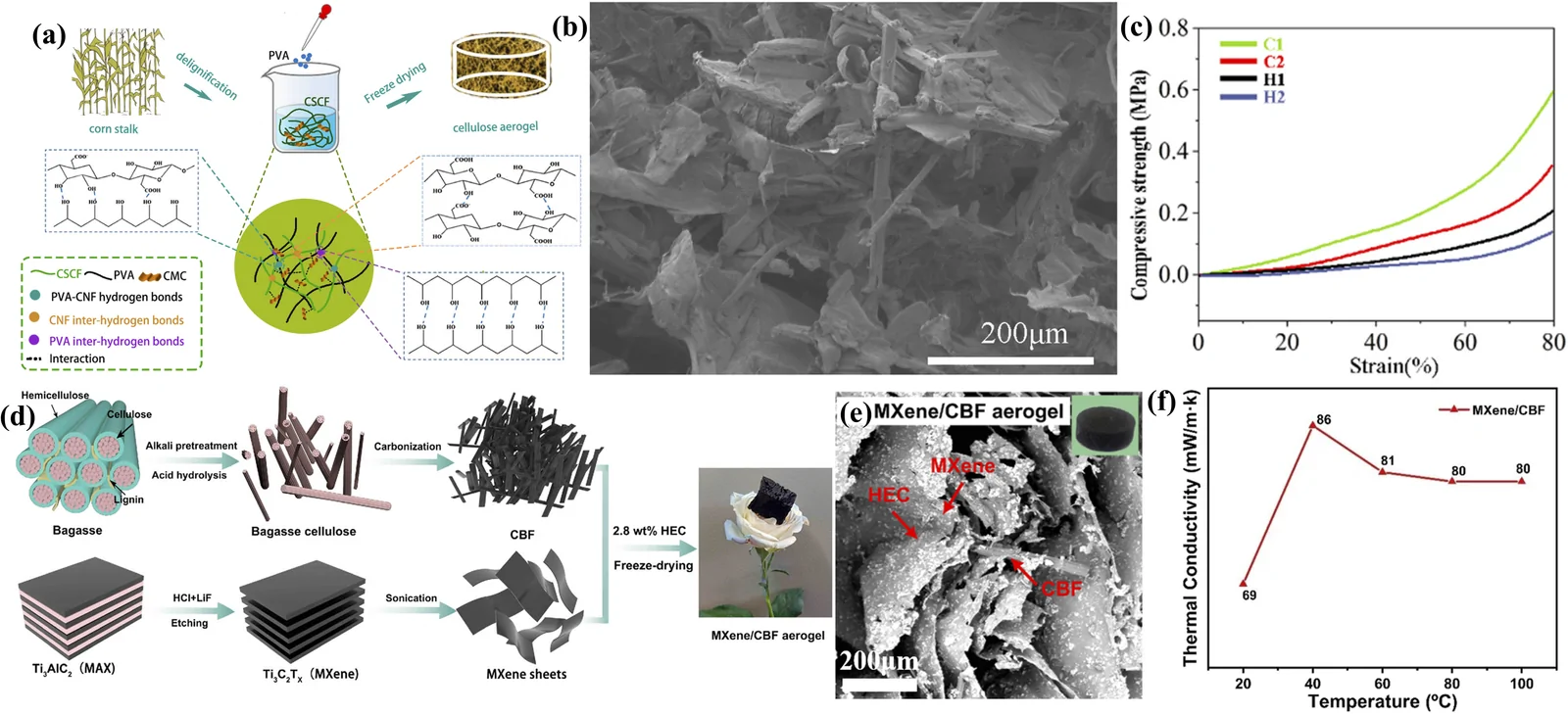
Structural construction and performance of agricultural-residue-derived fibrous aerogels. (a) Preparation of cornstalk-rind cellulose fiber/CMC/PVA aerogels and the proposed hydrogen-bonding interactions among the three components. (b) Cross-sectional SEM image showing cornstalk-derived cellulose fibers embedded within the porous CMC/PVA network. (c) Compressive stress–strain curves of aerogels prepared under different delignification and formulation conditions. (d) Preparation of carbonized bagasse fibers and Ti_3_C_2_T_*x*_ MXene nanosheets, followed by assembly into MXene/CBF aerogels using hydroxyethyl cellulose as a linker. (e) Photograph and cross-sectional SEM image showing the interconnected carbonized-fiber/MXene framework. (f) Temperature-dependent thermal conductivity of the MXene/CBF aerogel between 20 and 100 °C. (a–c) Reproduced with permission. Copyright 2024, MDPI AG.^49^ (d–f) Reproduced with permission. Copyright 2024, AIP Publishing.^50^

A different balance between mechanical reinforcement and thermal transport was obtained using bagasse. Zhu *et al.* removed hemicellulose and lignin from bagasse, carbonized the isolated fibers at 600 °C for 2 h, and assembled them with Ti_3_C_2_T_*x*_ MXene nanosheets using 2.8 wt% hydroxyethyl cellulose as a linker.^50^ The preparation route and resulting structure are presented in Fig. 2d and e. Hydroxyethyl cellulose maintained contact between the one-dimensional carbonized fibers and two-dimensional MXene sheets, producing an interconnected framework rather than densely stacked MXene layers. The aerogel reached a compressive stress of 1.04 MPa and a thermal conductivity of 69 mW m^−1^ K^−1^ at 20 °C. Its thermal conductivity increased to 86 mW m^−1^ K^−1^ at 40 °C and then decreased slightly to approximately 80 mW m^−1^ K^−1^ at 80–100 °C (Fig. 2f). Relative to the cornstalk-derived aerogel, the higher conductivity reflects the introduction of carbonized fibers and MXene, both of which reinforce the solid heat-transfer network. Because carbonization converts the cellulose-derived scaffold into a carbon network, this system is treated here as a structural comparator rather than as direct evidence for a polymer fibrous aerogel; its higher conductivity also illustrates the thermal penalty of reinforcing the solid heat-transfer pathway with carbonized fibers and MXene.

The hydroxyl-rich surface responsible for aqueous dispersion and interfiber bonding also makes cellulose sensitive to moisture. Adsorbed water replaces low-conductivity air, plasticizes the skeleton, and weakens reversible contacts. Yin *et al.* addressed this limitation by crosslinking cellulose-diacetate nanofibers *in situ* with a blocked isocyanate.^55^ The resulting aerogels retained a porosity of 99.25–99.36% and a thermal conductivity of 27.2–28.4 mW m^−1^ K^−1^, while exhibiting a water-contact angle of 162° and surviving 20 000 bending cycles. Chen *et al.* used hydrophobic modification in a nanocellulose aerogel and obtained a thermal conductivity of 23.9 mW m^−1^ K^−1^ together with thermal-barrier performance during exposure to 1300 °C.^56^ Cotton-fiber/silica composites and surface-modified cellulose aerogels provide related routes for combining hydrophobicity with mechanical recovery.^57,58^ The objective of these treatments is not simply to maximize contact angle, but to stabilize fiber contacts without sealing the pore network. Vapor sorption, humid-state thermal conductivity, and cyclic wetting durability are more meaningful measures of moisture resistance than a static water-contact angle alone.

Beyond moisture stability, fire safety is another critical consideration for cellulose-based fibrous aerogels, particularly in high-temperature or flame-exposed environments. The relatively high combustibility and limited thermal stability of cellulose compared with aromatic high-performance polymers necessitate the incorporation of mineral or char-forming phases to protect the cellulose-rich fibrous skeleton. For example, MOF-modified lignocellulosic aerogels employ thermally stable inorganic phases to reinforce pore walls, promote protective residue formation, and suppress heat and mass transport during combustion.^60^ A hydroxyapatite-nanowire/aramid-nanofiber/nanocellulose aerogel achieved an overall thermal conductivity of 26.02 mW m^−1^ K^−1^ and a radial conductivity of 22.89 mW m^−1^ K^−1^, showing that a sufficiently porous inorganic network can improve thermal stability without necessarily creating a dense thermal bridge.^61^ The amount and continuity of the protective phase remain critical: the fire-protective phase should stabilize the cellulose network while avoiding continuous solid pathways that increase conduction.

Regenerated-cellulose fibers illustrate the competing effects of crosslinking and environmental exposure. The regeneration rate governs skin formation, radial shrinkage, pore continuity, and humidity response.^79,80^ Jiang *et al.* used 1.5 wt% methylene diphenyl diisocyanate to reinforce cellulose aerogel fibers, obtaining a tensile strength of 27.7 MPa and a Young's modulus of 735 MPa.^66^ At 50–60% relative humidity, the resulting fabric provided insulation comparable to cotton wadding at only one-fifth of its thickness. At 98% relative humidity, however, its thermal conductivity increased to approximately 0.09 W m^−1^ K^−1^. Strengthening the fiber skeleton improves dimensional retention but does not eliminate hydration-dependent heat transfer.

Recent work further shows that cellulose-based aerogels are moving from composition-level modification toward explicit control of interface chemistry, pore orientation, and spatial gradients. PVTMS-modified TEMPO-oxidized cellulose nanofiber aerogels combine silane-derived interfacial regulation with directionally controlled drying to create strongly anisotropic networks.^81^ A related TOCNF/PVTMS/LDH aerogel integrates a biomimetic tracheal pore architecture with organosilicon/inorganic stabilization,^82^ while sequential pre-freezing produces Janus CNF/PET microfiber cryogels with porosity above 96% and mechanically persistent double-network architectures.^83^ These studies illustrate a transition from composition-based modification toward deliberate control of network topology; their consequences for pore-mediated and directional heat transport are analyzed in Sections 4.2 and 4.3.

### 2.3 Other bio-derived fibrous systems

The cornstalk and bagasse systems demonstrate how chemical treatment changes the composition and continuity of an agricultural-fiber skeleton. Native hollow or branched fibers provide a complementary strategy in which the original biological geometry is retained instead of being extensively fibrillated or carbonized. Directionally freeze-dried down-fiber/CNF aerogels with a density of approximately 40 mg cm^−3^ recovered completely after 90% compression because the branched down fibers redistributed stress along the pore walls and prevented localized fracture of the CNF skeleton.^51^ When natural down fibers formed a three-dimensional reinforcing framework in silk-fibroin aerogels, the composite reached a thermal conductivity of approximately 34 mW m^−1^ K^−1^.^52^ Kapok fibers provide a clearer example of morphology-assisted thermal regulation because their hollow lumens interrupt continuous solid conduction and retain air within the fiber interior. A calcium-alginate/kapok-fiber/CNT aerogel achieved a density of 0.027 g cm^−3^ and a thermal conductivity of 24 mW m^−1^ K^−1^ despite the incorporation of conductive carbon nanotubes.^53^ By comparison, glutaraldehyde-crosslinked CMC aerogels reinforced with micron-scale down-feather fibers had densities of 0.0389–0.0428 g cm^−3^, a porosity of approximately 97%, and a thermal conductivity of 60.12 mW m^−1^ K^−1^, although their compressive stress at 80% strain increased by approximately 75%.^54^ The benefit of a natural hollow fiber depends on whether its lumen remains open, how uniformly the fibers are distributed, and whether the surrounding binder forms additional solid bridges. Loofah-derived carbon aerogels provide a complementary morphology-preservation benchmark in which PVTMS-assisted stabilization and pyrolysis retain the native vascular channels.^84^ Because this framework is carbonized, it is used here only as a bio-derived architectural comparator; its direction-dependent transport is discussed in Section 4.3.

Other bio-derived polymers broaden the available gelation and junction chemistries. Alginate enables rapid ionic crosslinking, chitosan provides amine-rich interactions and char-forming chemistry, silk fibroin forms cohesive protein networks, and agarose or starch supplies physical gelation pathways. In starch/clay aerogels, cellulose nanofibrils allowed the starch concentration to be reduced from 4 to 2 wt%, decreasing the density from 0.12 to 0.05 g cm^−3^ and the thermal conductivity from 48.3 to 41.5 mW m^−1^ K^−1^.^85^ The nanofibrils were effective because they reduced the amount of dense starch matrix required to maintain the pore walls. Graphene/chitosan networks similarly strengthened the porous skeleton and increased heat-transfer tortuosity.^86^ Alginate and chitosan also illustrate distinct fire-control routes: alginate/Al_2_O_3_-fiber aerogels use ionic network formation together with mineral reinforcement to support the porous skeleton during combustion,^59^ whereas PVA/APP/chitosan aerogels promote char formation and suppress heat release as the APP–chitosan fraction increases.^62^ At excessive loading, however, densification increases solid-phase conduction. These examples show that each bio-derived polymer should be evaluated according to its own gelation chemistry and degradation pathway rather than grouped by origin.

Continuous aerogel fibers introduce additional structural control because coagulation and secondary crosslinking determine skin formation, radial shrinkage, pore continuity, and moisture transport. Du *et al.* used thermoreversible agarose gelation to stabilize a silk-fibroin network during continuous fiber formation, followed by hydrophobic chemical-vapor deposition.^87^ The resulting fibers had densities below 0.153 g cm^−3^, porosities of 91.1–94.7%, specific surface areas above 254.0 m^2^ g^−1^, and a water-contact angle of 136.8°. They recovered after 60% radial compression and retained thermal-insulation performance between −60 and 140 °C. Reversible gelation preserved a deformable porous network, while the surface treatment reduced direct water uptake without replacing the internal pore structure with a dense coating.^88^

Fig. 3 deliberately juxtaposes two continuous aerogel-fiber routes from different bio-derived classes rather than treating them as a single material category. In the silk-fibroin/agarose system, thermoreversible gelation establishes the continuous fiber geometry, whereas vapor-phase hydrophobic modification stabilizes the external boundary (Fig. 3a). The infrared images confirm thermal shielding under different temperature conditions (Fig. 3b), and the tensile and radial-compression results show that the porous fibers sustain axial loading while accommodating large transverse deformation (Fig. 3c–e). In contrast, the silica/bacterial-cellulose system forms an interpenetrating inorganic network within a nanofibrous cellulose scaffold (Fig. 3f). Sai *et al.* reported a tensile strength of 5.4 MPa, a specific surface area of up to 606.9 m^2^ g^−1^, a density below 0.164 g cm^−3^, and a decomposition temperature of approximately 330 °C.^89^ The comparison highlights distinct network-forming mechanisms: physical gelation and protein-chain assembly in the former *versus* cellulose-fibril templating and inorganic network formation in the latter.

**Fig. 3:**
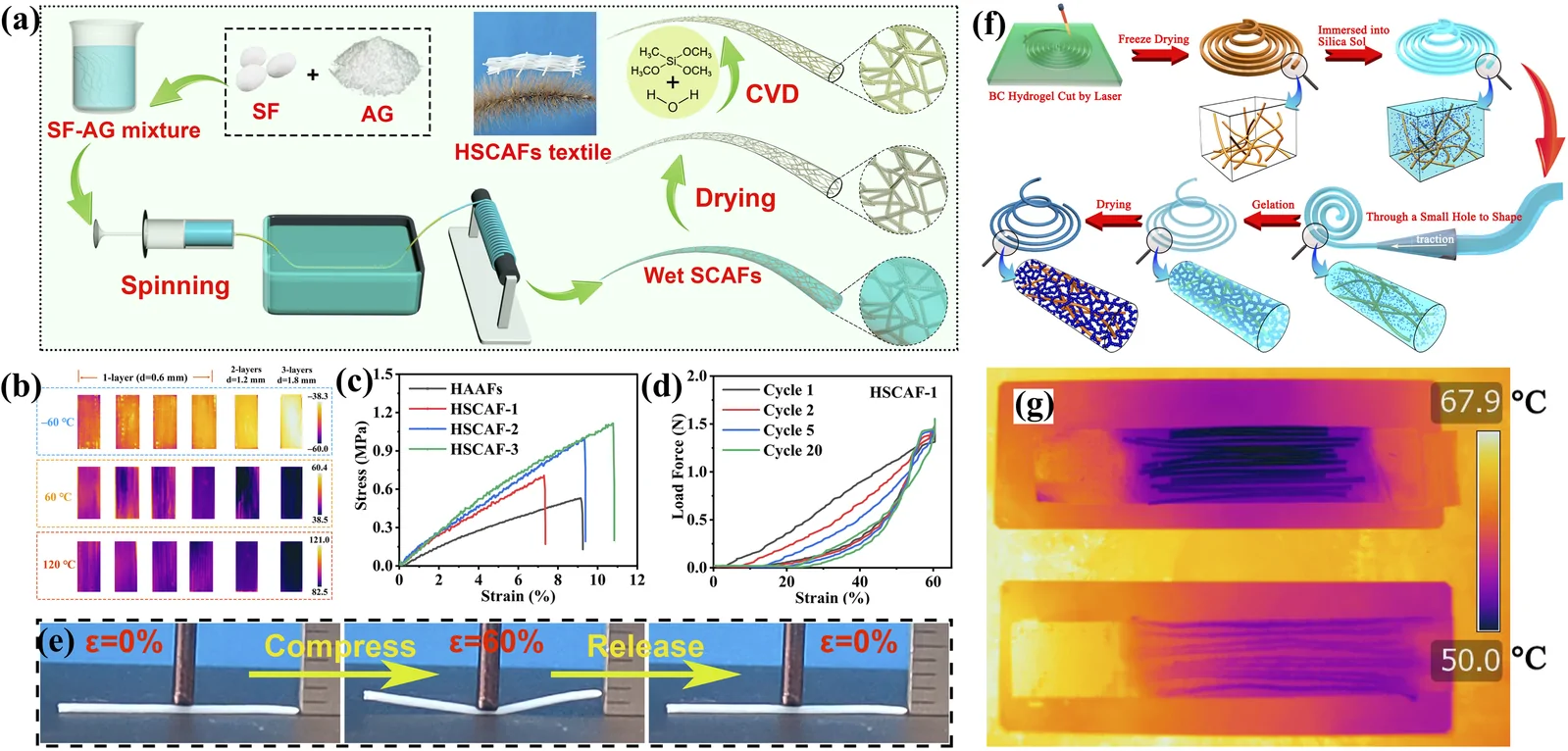
Structural design and thermal performance of continuous bio-derived composite aerogel fibers. (a) Continuous preparation and hydrophobic modification of silk-fibroin/agarose composite aerogel fibers. (b) Infrared thermal images of the fibers under different temperature conditions. (c) Tensile stress–strain behavior. (d) Cyclic radial-compression performance. (e) Deformation and recovery of an individual fiber at 60% radial strain. (f) Preparation of silica/bacterial-cellulose composite aerogel fibers through the formation of an interpenetrating organic–inorganic network. (g) Infrared images showing the thermal-insulation performance of the resulting fiber assemblies. (a–e) Reproduced with permission. Copyright 2024, MDPI AG.^87^ (f and g) Reproduced with permission. Copyright 2021, MDPI AG.^89^

Taken together, these systems should be classified by the mechanism that creates and stabilizes the network rather than by biological origin alone. Cellulose relies primarily on fibrillation and hydroxyl-mediated interfiber association; alginate on ionic gelation; chitosan on amine-rich association and char-forming chemistry; silk fibroin on protein-chain assembly; agarose and starch on physical gelation; and natural hollow fibers on inherited morphology.^47,52,85–88,90–93^ These distinctions govern moisture uptake, thermal degradation, junction reversibility, and the structural changes that accompany service exposure, and require material-specific design and validation criteria.

### 2.4 Aramid and PBO fibrous systems

Aramid nanofibers (ANFs) combine a rigid aromatic backbone with strong intermolecular hydrogen bonding, giving them higher thermal stability, lower creep, and better flame resistance than most polysaccharide fibers. However, these molecular interactions also make fibrillation and subsequent assembly difficult to control. Alkali-assisted deprotonation separates macroscale aramid fibers into negatively charged nanofibers, whereas reprotonation restores hydrogen bonding and converts the dispersion into a connected solid network. Hu *et al.* regulated this transition using proton donors and obtained aerogel membranes with a porosity of 99.53% and a tensile strength of 11.8 MPa, which was 16.5 times higher than that of the unregulated network. The membranes also maintained thermal resistance above 500 °C.^15^ Acid-assisted assembly can produce even stronger junctions. Wu *et al.* combined directional freezing with acid-induced crosslinking to obtain aligned aramid aerogels with a thermal conductivity of 15.8 mW m^−1^ K^−1^, a toughness of 1.3 MJ m^−3^, and a fracture energy of 7.36 kJ m^−2^; the materials provided insulation from −130 to 300 °C and resisted direct combustion for 20 min.^16^ Reprotonation must be sufficiently slow to preserve an open network but sufficiently extensive to establish mechanically effective contacts.^42,43^

Fiber morphology provides another means of controlling network formation. Branched ANFs form more intersections than linear nanofibers and reach mechanical percolation at very low solid contents. Li *et al.* prepared branched-ANF aerogels with a density of 0.0081 g cm^−3^, an average open-pore size of 21.5 nm, and a thermal conductivity of 0.026 W m^−1^ K^−1^.^17^ Despite the low density, the aerogel sustained a compressive stress of 825 kPa at 80% strain and remained thermally stable up to approximately 530 °C. Steam explosion offers a less chemically intensive route to similar fibrillar building blocks. Li *et al.* converted PPTA pulp into branched microfibrils that could be dispersed in water and directionally freeze-cast.^69^ Interfiber entanglement produced an aerogel with a specific surface area of 134.32 m^2^ g^−1^, a thermal conductivity of 0.03137 W m^−1^ K^−1^, and thermal stability above 500 °C. The slightly higher conductivity than that of fully nanoscale ANF networks reflects the coarser fibrils and larger solid pathways, but the aqueous processing route reduces dependence on prolonged deprotonation. Complete nanofiber individualization is not always necessary; partially fibrillated and branched structures can provide sufficient connectivity with simpler processing.

After association has been established, pore geometry determines whether the network can tolerate repeated deformation. Foldable and stretchable ANF aerogels show that reversible fiber bending and sliding can accommodate large macroscopic strains without fracturing the aromatic skeleton.^94^ Directional freezing further organizes ANFs into hierarchical cellular networks: Shi *et al.* achieved a thermal conductivity of 23.7 mW m^−1^ K^−1^ by combining larger deformation-accommodating cells with finer pores that restrict gas conduction.^95^ Multistage and layered pores similarly distribute compressive stress and lengthen heat- and sound-transfer pathways.^96^ Secondary polymers or fibers can assist this assembly, but their function depends on location. PVA-regulated ANF aerogels reached a density of 9.98 kg m^−3^ and a thermal conductivity of 25.78 mW m^−1^ K^−1^ because PVA moderated rapid ANF aggregation and stabilized fiber contacts.^97^ In foam-formed aramid aerogels, bacterial cellulose nanofibers primarily reinforce the skeleton through physical entanglement, whereas carboxylated cellulose provides stronger interfacial interactions and more effectively regulates pore-wall formation.^98^ The secondary phase should control association or reinforce discrete contacts rather than form a continuous matrix.^99^

PBO follows a fundamentally different route to nanofibrillation and gel formation. Whereas aramid-network regeneration is governed largely by deprotonation/reprotonation and restoration of amide-mediated hydrogen bonding, the rigid, highly conjugated benzobisoxazole backbone of PBO is insoluble in common solvents and exhibits strong interchain association. Strong protonic acids protonate benzobisoxazole nitrogen sites, attenuate interchain π–π interactions, and introduce electrostatic repulsion, enabling macroscale PBO fibers to disassemble into PBO nanofibers (PNFs); subsequent deprotonation and solvent exchange restore interchain association and permit three-dimensional assembly.^100^ Earlier PBO aerogel studies used the thermally persistent PNF skeleton together with secondary networks to stabilize mechanically weak contacts, including dual-crosslinked polybenzoxazine/PNF structures and continuously processable PBO aerogel fibers.^101–103^ The defining processing distinction is molecular: protonation provides access to the nanoscale PBO building block, whereas post-protonation association and junction stabilization determine whether that building block is retained as an open, mechanically persistent network.

Functional fillers introduce a further trade-off. Carbonization improves temperature resistance and structural persistence under extreme conditions but replaces the electrically insulating aromatic network with a more conductive carbon skeleton.^104^ CNTs, liquid-metal@MoS_2_ particles, and reduced graphene oxide provide dielectric loss, interfacial polarization, or infrared attenuation, but they can also increase solid conduction when they form continuous pathways.^105–107^ For example, ANF-reinforced RGO aerogels achieved a minimum microwave reflection loss of −56.5 dB, an effective absorption bandwidth of 7.0 GHz, and a compressive stress of 78.8 kPa at 70% strain, but their thermal conductivity increased to approximately 49.18 mW m^−1^ K^−1^.^107^ Liquid-metal@MoS_2_/ANF aerogels similarly reached a reflection loss of −36.9 dB and an effective absorption bandwidth of 3.68 GHz.^106^ These values confirm that multifunctionality is obtained at a measurable thermal cost. Conductive fillers should remain isolated or spatially confined unless electromagnetic performance is the primary objective.^108^

Processing intensity and material cost remain important limitations for both aromatic nanofiber families, but the origin of the processing penalty differs. ANF assembly depends on deprotonation, dispersion, and reprotonation-driven recovery of hydrogen-bonded contacts, whereas PBO requires strong-acid protonation to overcome the low solubility and strong association of its rigid heteroaromatic backbone.^42,43,100^ Upcycling waste heterocyclic aramid fibers reduces precursor demand but does not eliminate the solvents and reagents required for fibrillation,^109^ while recyclable aramid aerogel fibers with homogeneous nanoscale structures have reached a tensile strength of approximately 30 MPa and a tensile strain of 30%.^110^ Neither aramid nor PBO should be regarded as intrinsically low-conductivity in every form; their insulating performance ultimately depends on retaining a sparse fibrillar skeleton, discontinuous transverse contacts, and tortuous heat-transfer paths after drying and service.

### 2.5 Polyimide and polyamide-imide fibrous systems

Polyimides (PIs) provide a broad molecular-design space because the rigidity, symmetry, polarity, and steric configuration of dianhydride and diamine units can regulate chain packing, imidization shrinkage, thermal stability, and pore retention. However, these molecular advantages contribute to insulation only when they are translated into an appropriate fibrous architecture. Directional freezing of all-aromatic PI precursors produced anisotropic cellular networks that retained their structure after high-temperature exposure; for example, the temperature measured 25 mm from the hot end of a unidirectionally frozen PI aerogel was only 76.3 °C after exposure to 300 °C for 20 min.^33^ The axial thermal conductivity was nevertheless higher than the radial value, showing that alignment improves mechanical integrity but can also establish preferential solid-conduction pathways. Hierarchical cellular crosslinking provides an alternative means of stabilizing the network. Crosslinked PI nanofibrous aerogels reached a thermal conductivity of 33.2 mW m^−1^ K^−1^ while maintaining high porosity and mechanical stability.^34^ Multilayer PI aerogels further separated load-bearing, insulating, and sensing functions between different layers, thereby avoiding uniform densification of the entire structure.^35^ The desirable architecture is not the most highly aligned or densely crosslinked network, but one that combines curved or cellular fibers with controlled transverse contacts.

At the molecular level, rigid–flexible coupling can suppress shrinkage without eliminating the chain mobility required for gel formation. Hu *et al.* balanced rigid aromatic segments and flexible linkages to improve dimensional stability and thermal insulation under extreme conditions,^36^ while Mosanenzadeh *et al.* varied the ratio of two dianhydrides to regulate backbone rigidity, pore formation, and high-temperature performance.^37^ Sterically demanding “paddle-shaped” diamines further inhibited close chain packing during imidization and preserved the porous structure.^38^ The resulting relationship between molecular structure and insulation is illustrated in Fig. 4a–d. The BPDA-6FAPB aerogel exhibits an open cellular structure on its top surface and cross-section, whereas the lower surface is comparatively compact and directionally organized (Fig. 4a), indicating that molecular packing and processing-induced shrinkage jointly determine spatial pore uniformity. The thermal conductivities of PMDA-ODA, BPDA-6FAPB, and copolyimide aerogels were 0.098, 0.086, and 0.056 W m^−1^ K^−1^, respectively (Fig. 4b).^39^ The copolyimide reduced thermal conductivity by approximately 43% relative to PMDA-ODA. It also maintained the lowest surface temperature during 100 s of heating, as confirmed by the temperature–time curves and infrared images in Fig. 4c and d. An appropriate combination of rigid and flexible units can increase free volume, preserve air-filled pores, and interrupt continuous solid-phase heat-transfer pathways.

**Fig. 4:**
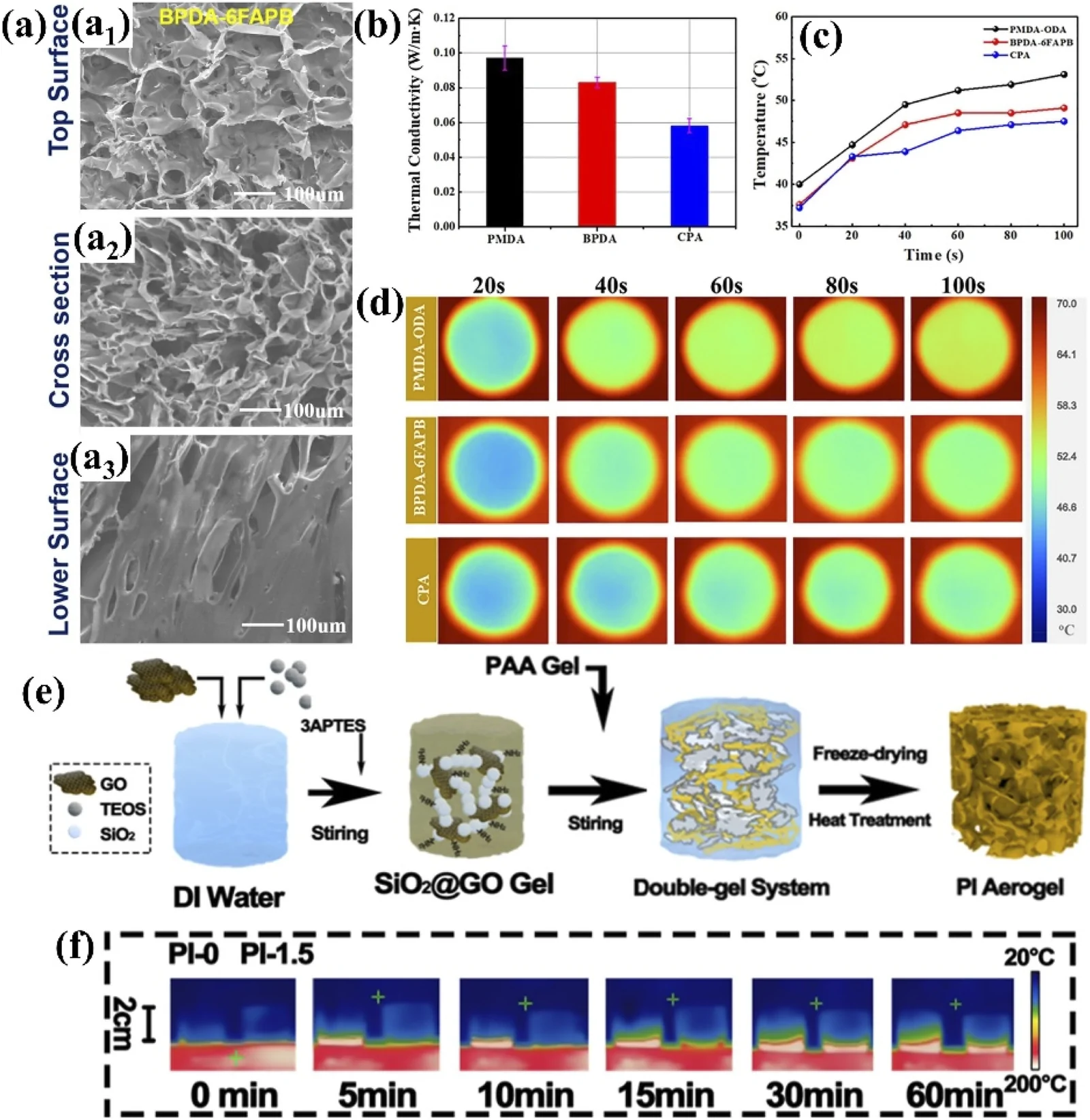
Molecular-structure and double-network regulation of polyimide aerogels. (a) SEM images of the top surface (a_1_), cross-section (a_2_), and lower surface (a_3_) of the BPDA-6FAPB aerogel; (b) thermal conductivities of PMDA-ODA, BPDA-6FAPB, and copolyimide aerogels; (c) surface-temperature evolution and (d) corresponding infrared thermal images during 100 s of heating. (e) Preparation of the amino-functionalized SiO_2_@GO/poly(amic acid) double-gel system followed by freeze-drying and thermal imidization; (f) time-resolved infrared images of PI-0 and PI-1.5 placed on a 200 °C heating stage for 60 min.^78^ (a–d) Reproduced with permission. Copyright 2022, The Institution of Engineering and Technology.^39^ (e and f) Reproduced with permission. Copyright 2022, MDPI AG.^78^

For polyamide-imide (PAI) and PI aerogel fibers, network topology becomes particularly important because the material must withstand tensile deformation as well as compression. A thermally crosslinked semi-interpenetrating PAI/meta-aramid micro/nanofibrous network achieved a minimum thermal conductivity of 26.22 mW m^−1^ K^−1^.^40^ The crosslinked PAI phase stabilized fiber contacts, whereas the meta-aramid framework maintained the open pore structure, demonstrating that interpenetration is beneficial when bonding is concentrated around existing fibers rather than throughout the pore volume. Prefabricated long PI fibers produced an even greater mechanical improvement: the resulting core-reinforced aerogel fibers exhibited tensile strengths above 150 MPa and retained their mechanical performance from −100 to 300 °C.^41^ Topological PAI/PU aerogel fibers similarly combined multilevel hollow pores with entangled polymer chains, reaching a breaking strength of 94.5 MPa and a breaking strain of 20%.^74^ Continuous reinforcing fibers and topological entanglements can carry tensile loads more efficiently than homogeneous matrix thickening, thereby reducing the usual conflict between mechanical strength and low thermal conductivity.

Organic–inorganic double networks provide another means of stabilizing PI aerogels during drying, imidization, and high-temperature exposure. An integrated PI/SiO_2_ network containing up to 60% inorganic component tolerated 500 compression cycles at 50% radial strain, exhibited a thermal conductivity of 21.2 mW m^−1^ K^−1^, and resisted a 1200 °C flame without disintegration.^75^ A binary PI@SiO_2_ nanofibrous aerogel similarly achieved a thermal conductivity of 27.2 mW m^−1^ K^−1^ while retaining repeated-compression superelasticity.^76^ A polymer-dominant strategy was demonstrated using thermally crosslinked, nanofiber-reinforced PI aerogels. At a density of 26 mg cm^−3^ and a porosity of 94.92%, these aerogels showed a thermal conductivity of 30.06 mW m^−1^ K^−1^ and only 7% permanent deformation after 1000 compression cycles.^77^ The construction and thermal effect of an organic–inorganic double-gel system are further illustrated in Fig. 4e and f. Amino-functionalized SiO_2_@GO wet gel was incorporated into the poly(amic acid) gel, followed by freeze-drying and thermal imidization to form an interconnected PI/SiO_2_/GO network (Fig. 4e). The optimized PI-1.5 aerogel had a density of 0.0441 g cm^−3^, a thermal conductivity of 0.0309 W m^−1^ K^−1^, a specific modulus of 46.19 m^2^ s^−2^, and a water-contact angle of 131.55°.^78^ When PI-0 and PI-1.5 were placed on a 200 °C heating stage, PI-1.5 showed slower heat penetration throughout the 60 min test (Fig. 4f), confirming that the double network improved thermal resistance without completely filling the pores. A secondary network is most effective when it stabilizes fiber contacts and pore walls; excessive inorganic continuity would instead increase density and solid-phase conduction.

Although PI is generally less hygroscopic than cellulose, moisture resistance cannot be inferred from polymer identity or contact angle alone. Moisture-resistant PI aerogel membranes achieved a thermal conductivity of 35.4 mW m^−1^ K^−1^ while retaining low dielectric properties under harsh conditions,^111^ whereas PI/silica double-network fibers used the inorganic network to restrict swelling and structural rearrangement in hot and humid environments.^112^ A more direct comparison was provided by PVDF/PI nanofibrous aerogels. Their thermal conductivity increased from 31.0 mW m^−1^ K^−1^ under ambient conditions to only 48.6 mW m^−1^ K^−1^ at 80 °C and 100% relative humidity, whereas commercial silica aerogel reached 120.5 mW m^−1^ K^−1^ under the same conditions.^113^ Low surface energy must operate together with a mechanically persistent pore network to prevent moisture-induced deterioration.

The versatility of PI also means that its structural role must be identified before different systems are compared. In PI/aramid composites, PI infiltration reduced thermal conductivity from 0.047 to 0.031 W m^−1^ K^−1^ while strengthening interfiber contacts.^114^ Multidimensional aramid/PI networks used aramid nanofibers as reinforcement and PI as the thermally stable porous phase,^115^ whereas wood/PI aerogels retained the hierarchical channels of wood while improving fire and water resistance.^116^ PAN-nanofiber-reinforced PI aerogels formed fiber–pore interpenetrating structures that combined thermal insulation with filtration and acoustic absorption.^117^ Fluorinated PI/paraffin composites adopted a sandwich configuration to separate thermal-barrier and phase-change functions.^118^ Consequently, PI may function as the primary skeleton, binder, coating, reinforcing phase, or continuous matrix, and these configurations should not be evaluated as equivalent materials. Meaningful comparison requires reporting the load-bearing skeleton, imidization degree, fiber fraction, crosslink density, directional shrinkage, and continuity of the secondary phase. For thermal insulation, the preferred configuration is generally a sparse but dimensionally stable fibrous skeleton with localized contacts and persistent pores rather than a pore-filling PI matrix.

### 2.6 Other polymeric fibrous systems

Beyond cellulose, aramid, PBO, PI, and PAI, other polymers provide useful processing windows and functions. PVA and alginate support low-cost aqueous shaping and ionic or covalent multinetworks. Bai *et al.* developed multinet PVA/alginate fibers,^119^ while organosilicon-modified PVA and flame-retardant PVA/chitosan systems addressed moisture and fire limitations.^120,121^ Their principal liabilities remain humidity sensitivity and restricted high-temperature stability; crosslinking must strengthen junctions without converting a fibrous network into a dense film.

Melt- or solution-processable thermoplastics offer hydrophobicity, weldable contacts, and scalable fiber production. Tan *et al.* produced flexible superhydrophobic PLA hollow-fiber aerogels,^122^ Do *et al.* developed polypropylene-fiber aerogels,^123^ and Chen *et al.* used solubility–p*K*_a_ coupling to tailor nanoporous nylon aerogels.^124^ Polyurea-reinforced cellulose fibers provide a related strategy in which a ductile phase protects a fragile skeleton.^125^ These systems are attractive for clothing, packaging, transportation, and buildings, but creep, melting, and flammability impose application-specific upper-temperature limits.

Char-forming thermosets, including polybenzoxazine and phenolic resins, become advantageous when fire integrity or ablation resistance dominates. Fiber-reinforced polybenzoxazine (Fig. 5a–d), *in situ* polybenzoxazine/silica, and quartz- or carbon-fiber-reinforced phenolic aerogels form persistent carbonizable networks.^126–129^ Their rigid residues improve barrier integrity but may increase brittleness and solid conduction. Polymerization should be confined to junctions, thin skeletal coatings, or graded hot-face regions rather than uniformly filling the pore volume.

**Fig. 5:**
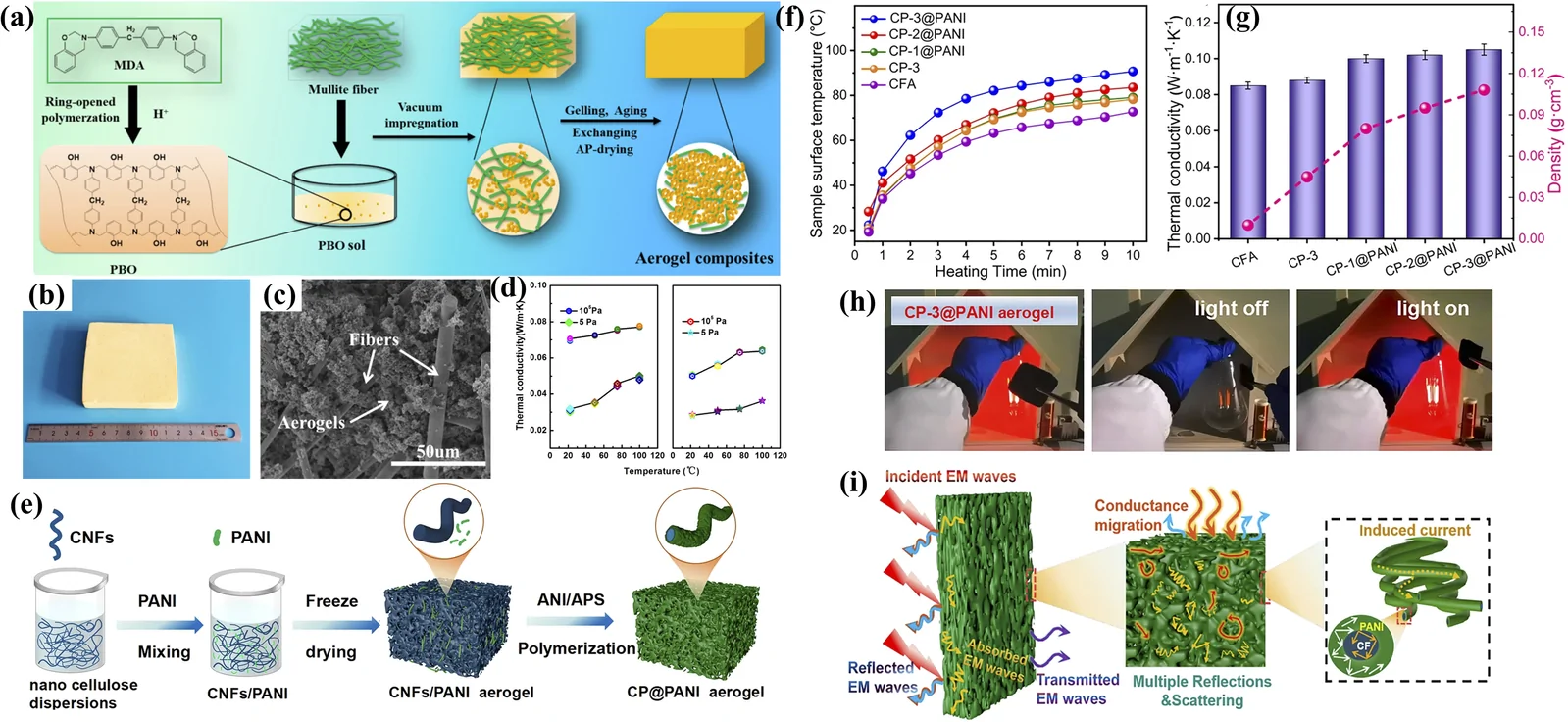
(a) Synthesis and structural composition of fiber-reinforced PBO aerogel composites; (b) photograph and (c) SEM image of the composites; (d) thermal conductivity of the composites and the PBO aerogel at 5 and 10^5^ Pa and at 22, 50, 75, and 100 °C. (e) Preparation of CP-3@PANI aerogels; (f) time-dependent surface temperature; (g) thermal conductivity and density; (h) electromagnetic-shielding demonstration; (i) proposed EMI-shielding mechanism, including the incident, reflected, absorbed, and transmitted fields. (a–d) Reproduced with permission. Copyright 2020, MDPI AG.^126^ (e–i) Reproduced with permission. Copyright 2024, Springer Nature.^131^

Hydrophobic and electrically active polymers introduce functions that a passive low-density skeleton cannot provide. PVDF can protect pore surfaces from moisture, whereas conductive polymers, carbon, MXene, or graphene enable heating, sensing, or electromagnetic response (Fig. 5e–i).^53,113,130,131^ These additions are beneficial only when spatially confined: a discontinuous functional skin or separated pathway can preserve passive insulation, whereas uniform percolation creates thermal bridges and hot spots. Spatial separation of functions is a more general design rule than the selection of any particular filler.

High-performance melt-processable thermoplastics such as PPS are attractive because they combine chemical resistance, favorable flame behavior, and compatibility with scalable ultrafine-fiber production. Experimentally, Yu *et al.* demonstrated an industrial sea-island melt-spinning route in which PPS ultrafine fibers were freeze-shaped into corrugated aerogel felts with a density of 6.4 mg cm^−3^, a thermal conductivity of approximately 28.0 mW m^−1^ K^−1^, large-deformation flexibility, and more than 10 000 compression cycles.^20^ This evidence establishes the feasibility of scalable PPS fibrous felts, but it does not demonstrate all-PPS self-supporting aerogel monoliths produced by alternative junction chemistries. Surface activation, sparse point welding, low-loading sol–gel bridges, and freeze-assisted assembly are proposed here only as testable future routes, not as established PPS processing strategies. Their viability must be evaluated against the central trade-off for inert thermoplastic fibers: sufficient junction formation is needed to prevent collapse, whereas excessive welding or continuous bridging would enlarge solid contacts and erode the interfacial resistance required for low thermal transport.

Table 1 consolidates these selection principles. No polymer family is universally superior: cellulose favors renewable aqueous processing but requires moisture and fire protection; aramid and PBO provide mechanically efficient, heat-resistant frameworks but demand intensive fibrillation; PI and PAI offer broad molecular and architectural tunability but require careful control of precursor conversion and shrinkage; and other thermoplastics or thermosets provide scalable processing, hydrophobicity, or fire integrity at the cost of creep, surface inertness, brittleness, or added solid conduction. Polymer selection should be based on the compatibility among molecular structure, fiber-forming route, junction chemistry, drying method, and service environment rather than on any single bulk property.

**Table 1:** Representative structure–property benchmarks for polymer fibrous aerogels and the corresponding material-selection implications

Polymer family	Representative architecture and processing	Density and porosity	Thermal conductivity (mW m^−1^ K^−1^)	Mechanical and service performance	Main limitation and design implication	Ref.
Cellulose-based fibrous systems	Covalently fused bacterial-cellulose nanofibers; aggregation-induced waste-cotton microfilaments; isocyanate-crosslinked cellulose-diacetate nanofibers	16.1 mg cm^−3^ and 98.8% porosity; 99.25–99.36% porosity for cellulose-diacetate aerogels	27.0 ± 0.2; 21.5 ± 0.2 for waste-cotton cellulose; 27.2–28.4 for cellulose diacetate	10 000 compression, 1000 folding, and 10 000 shear cycles; 91.26% height recovery after 100 compression cycles; water-contact angle 162° and 20 000 bending cycles	Renewable aqueous processing and abundant junction sites are advantageous, but moisture uptake and flammability remain limiting. Localized crosslinking, hydrophobic skins, and low-loading fire barriers are preferred to bulk densification	14, 45 and 55
Aramid and PBO	Branched aramid nanofibers (BANFs); steam-exploded PPTA microfibrils; PVA-regulated ANF networks; protonation-derived PBO nanofibers and hydrogen-bond-stabilized PBO aerogels	8.1 mg cm^−3^ with a 21.5 nm mean open-pore size; 9.98 mg cm^−3^ for the PVA-regulated ANF aerogel	26.0 for BANFs; 31.37 for steam-exploded PPTA microfibrils; 25.78 for the PVA-regulated ANF aerogel	825 kPa at 80% compression and thermal stability to about 530 °C; recyclable aerogel fibers reached about 30 MPa tensile strength and 30% strain	Aramid relies mainly on deprotonation/reprotonation and recovery of hydrogen-bonded contacts, whereas PBO nanofibrillation requires protonation of the rigid benzobisoxazole backbone and subsequent stabilization of PNF junctions. Both require sparse, mechanically effective contacts to avoid continuous solid heat-transfer paths	17, 69, 97, 100, 101 and 110
Polyimide and polyamide-imide	Thermally crosslinked PI nanofibrous aerogels; integrated PI/SiO_2_ double networks; long-PI-fiber reinforcement; PAI/meta-aramid semi-interpenetrating networks	26 mg cm^−3^ and 94.92% porosity; 44.1 mg cm^−3^ for the PI/SiO_2_/GO double-gel aerogel	30.06 for PI; 21.2 for an integrated PI/SiO_2_ network; 26.22 for a PAI/meta-aramid network	Only 7% permanent deformation after 1000 compression cycles; 500 cycles at 50% strain and resistance to a 1200 °C flame; long-fiber-reinforced PI aerogel fibers exceeded 150 MPa from −100 to 300 °C	PI chemistry offers wide thermal and structural tunability, but imidization shrinkage and excessive crosslinking can increase solid conduction. Controlled conversion, curved fibers, and contact-localized secondary networks are required	40, 41, 75, 77 and 78
PVA, alginate, PVDF, PLA, PP, nylon, and related flexible polymers	Helical PVDF micro/nanofibers formed by phase-separation-assisted electrospinning; physically crosslinked PP-fiber aerogels; multi-network, hollow-fiber, and secondary-molding routes	PVDF sponge: 7.1 mg cm^−3^ and 99.6% porosity; PP-fiber aerogels: 24–54 mg cm^−3^ and 94.71–97.82% porosity	PVDF: 24.85 initially and 33.3 after 60 washing cycles; PP-fiber aerogels: 36–39	PVDF sustained 200% tensile strain and retained its architecture after 60 washes; PP-fiber aerogels showed a compressive modulus of 27.54–58.58 kPa and water-contact angles of 123.5–133.8°	These polymers offer scalable aqueous, solution, or melt processing, but humidity sensitivity, creep, melting, and flammability constrain service temperature. Multinetwork junctions, hollow fibers, and surface protection should be selected for the intended environment	70 and 123
Polybenzoxazine, phenolic, and char-forming systems	Quartz-fiber-reinforced polybenzoxazine aerogels; hybrid carbon/quartz-fabric-reinforced phenolic-silica aerogel composites	Reinforcement raises the solid fraction relative to ultralight nanofiber aerogels; reported values depend strongly on fiber content, pressure, and graded-surface design	Polybenzoxazine composite: 69 at 10^5^ Pa and 31 at 5 Pa; carbon/quartz-phenolic-silica composite: about 112	Polybenzoxazine composite: >80% compression, 20.69 MPa modulus, and LOI 32.5%. Phenolic-silica composite: 5.96–17.01 MPa compressive strength, 0.017 mm s^−1^ ablation rate, and an internal temperature below 100 °C at 80 mm depth when the surface exceeded 2000 °C	Char-forming networks preserve integrity during fire or ablation, but brittle residues and continuous resin paths increase conduction. Polymerization should be confined to junctions or graded hot-face regions	126 and 129
PPS high-performance thermoplastics	Industrial sea-island melt-spun PPS ultrafine fibers assembled by freeze shaping into corrugated lamellar aerogel felts	PPS aerogel felt: 6.4 mg cm^−3^; demonstrated at 94 × 105 × 1 cm scale	28.0 for the PPS ultrafine-fiber aerogel felt^20^	More than 25% tensile strain, 95% compression, 180° bending, and over 10 000 compression cycles; the aerogel felt carried a 1 kg load at 5 mm thickness^20^	Melt spinning offers scale, chemical resistance, and durable fibers, but inert surfaces hinder dispersion and junction formation. Surface activation, sparse point welding, sol–gel bridging, and freeze-assisted assembly remain proposed development routes for PPS and require direct experimental validation	20

## 3 Assembly of three-dimensional fibrous networks

Transforming polymer fibers or fibrils into an aerogel involves four coupled structural transitions: formation of the fibrous building blocks, three-dimensional organization in a liquid medium, fixation of load-bearing junctions, and removal of the pore liquid. These operations cannot be optimized independently. Fiber diameter and curvature determine the concentration required to reach percolation; dispersion controls whether those fibers form an open network or compact bundles; junction chemistry determines whether the wet architecture can resist capillary and handling stresses; and drying finally decides how much of the designed pore volume survives. The relevant processing output is not the appearance of the wet gel, but the retained architecture after drying and repeated service. This section evaluates representative studies by connecting the processing variable that was changed to the measured change in network topology, thermal conductivity, deformation recovery, and scale-up behavior.

### 3.1 Fiber formation, fibrillation, and dispersion

The structural potential of a fibrous aerogel is established before three-dimensional assembly begins. Mean fiber diameter alone is insufficient because percolation and pore stability also depend on aspect-ratio distribution, continuity, curvature, bundle fraction, crystallinity, and surface chemistry. Long, slender fibers lower the solid concentration needed to span the volume, but their large surface area increases friction and aggregation during mixing. Curved or branched fibers introduce geometric interlocking and extra load-transfer contacts, whereas straight fibers pack more efficiently and can create continuous solid-conduction paths. The processing objective is consequently to produce a fiber population that remains dispersible while retaining enough length and nonlinearity to form a mechanically connected network at low density.

Electrospinning illustrates how jet solidification can be used to program both the individual fiber and the macroscopic network. Conventional electrospinning deposits nearly straight fibers as a dense two-dimensional mat; obtaining a three-dimensional aerogel requires either redispersing the mat or arresting the jet before lamellar packing occurs. Wang *et al.* controlled the interaction between water molecules and the spinning solution to trigger rapid phase inversion while regulating charge density to generate multiple jets.^18^ The charged jets solidified as interlocking curly PI nanofibers rather than flat layers. The resulting aerogel had a density of 2.4 mg cm^−3^ and a thermal conductivity of 22.4 mW m^−1^ K^−1^, and retained mechanical robustness from −196 to 300 °C. The evidence supports a structural conclusion: curvature stores deformation through fiber bending and straightening, so elastic recovery can be introduced without increasing the solid fraction or adding a dense elastomeric binder.^71,72^

Fig. 6 illustrates three structural strategies for preserving pore architecture under deformation and thermal exposure. Porous helical PVDF fibers produced by phase-separation-assisted electrospinning formed a hierarchical network with a density of 7.1 mg cm^−3^ and a porosity of 99.6%, enabling 200% tensile strain through reversible coil opening (Fig. 6a).^70^ After 60 washing cycles, the thermal conductivity increased only from 24.85 to 33.3 mW m^−1^ K^−1^, while the water-contact angle remained approximately 140°, confirming that helical interlocking limited wet-induced structural collapse. Three-dimensional reaction electrospinning similarly preserved crimped ceramic fibers within a large-scale volumetric network (Fig. 6b–f).^73^ Fiber straightening accommodated tensile strains of up to 100%, and the network retained stable mechanical responses over 1000 cycles at 40% strain. Despite its low density of 6 mg cm^−3^, the ceramic aerogel exhibited a thermal conductivity of 22.8 mW m^−1^ K^−1^ and limited the back-side temperature to 121 °C after 10 min of exposure to a *ca.* 1300 °C flame. Branched aramid nanofibers provided a third mechanism by increasing junction connectivity and distributing stress across the pore walls (Fig. 6g–j).^17^ The resulting aerogel achieved a thermal conductivity of 26 mW m^−1^ K^−1^ at 8.1 mg cm^−3^ and sustained a compressive stress of 825 kPa at 80% strain. Helical opening, crimped-fiber straightening, and branch-mediated load transfer can preserve low-density porous networks under washing, repeated deformation, compression, and high-temperature exposure.

**Fig. 6:**
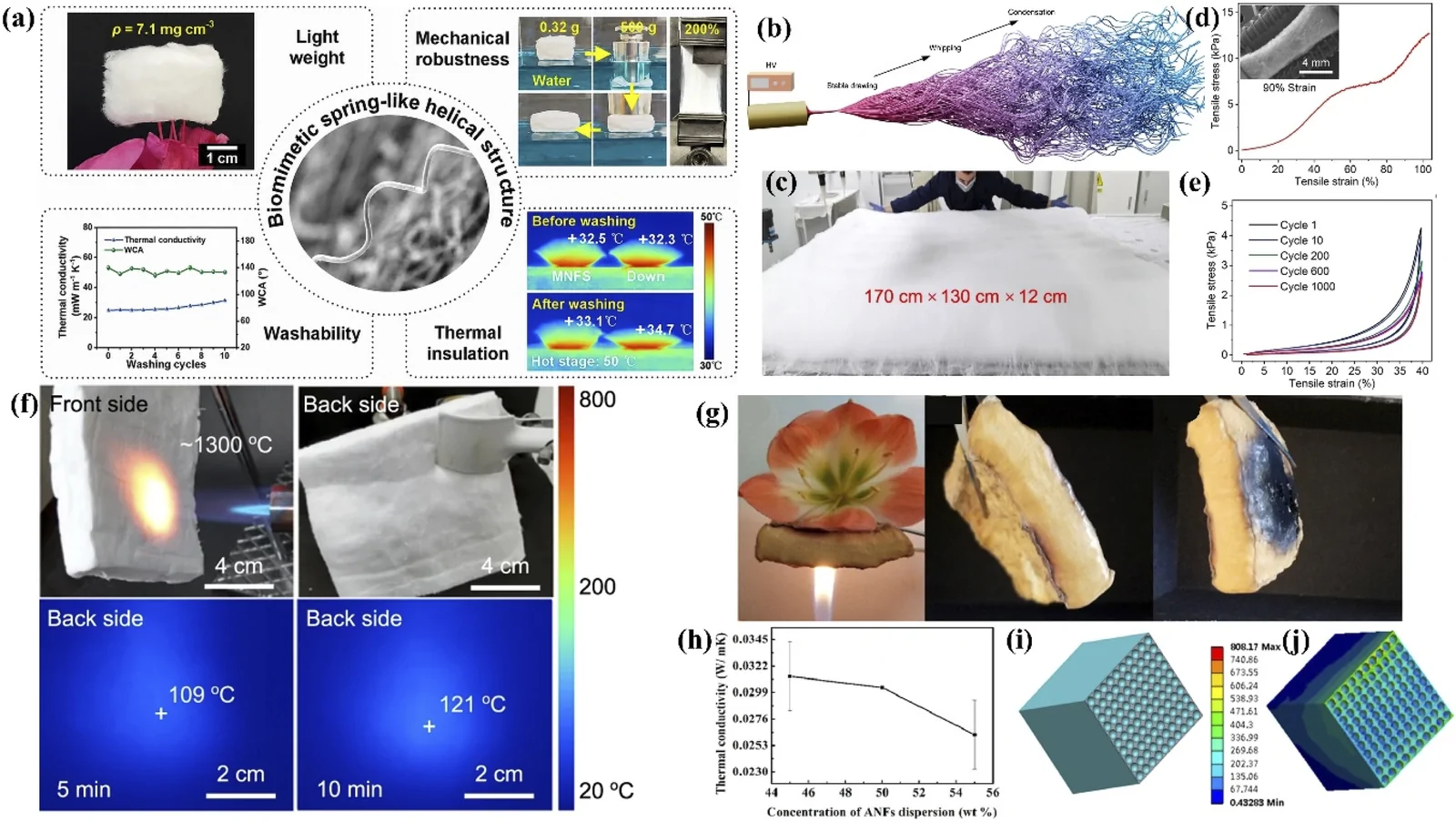
Fiber geometry and network connectivity established before drying. (a) Multiple-jet electrospinning of porous spring-like PVDF micro/nanofibers and their direct entanglement into MNFS; the panel combines the 7.1 mg cm^−3^ density, 200% tensile deformation, underwater compression, washing-dependent thermal conductivity and water-contact angle, and hot-stage comparison with down. (b) Three-dimensional reaction electrospinning, in which rapid sol–gel condensation arrests crimped ceramic jets and interweaves them into a volumetric precursor; (c) 170 × 130 × 12 cm^3^ precursor ICCA; (d) tensile stress–strain curve and *in situ* SEM image at 90% strain, showing straightening and reorientation of the crimped fibers; (e) 1000 tensile–release cycles at 40% strain; (f) optical and infrared images during *ca.* 1300 °C flame exposure, with back-side temperatures of 109 and 121 °C after 5 and 10 min, respectively. (g) BANF concentration-dependent thermal conductivity; (h) orchid-protection test during 5 min of flame exposure; (i) compression to 80% strain and the corresponding cheese-like open-pore model; (j) finite-element stress distribution showing localization at interconnected pore-wall junctions. (a) Reproduced with permission. Copyright 2022, Springer Nature.^70^ (b–f) Reproduced with permission. Copyright 2022, Springer Nature.^73^ (g–j) Reproduced with permission. Copyright 2021, Wiley-VCH GmbH.^17^

### 3.2 Three-dimensional assembly and pore templating

Once a processable fiber population has been established, assembly determines how solids, contacts, and voids are distributed in three dimensions. Random gelation, crushed-ice casting, directional freezing, foaming, scaffold-directed organization, and additive manufacturing impose different spatial constraints and generate different combinations of isotropy, pore connectivity, density uniformity, and directional mechanics. The correct comparison is not simply between named processing methods, but between the pore-size distribution and fiber orientation that each method retains at the dimensions and cooling rates required for manufacture.

Random or quasi-isotropic assembly is attractive for components exposed to multidirectional loads and heat flux, but freezing normally produces aligned columnar pores. Li *et al.* decoupled nucleation from crystal growth by freezing a nanofiber slurry on a rotating cryogenic drum, crushing the ice, mixing the equiaxed particles with additional slurry at a 5 : 1 mass ratio, recasting at −20 °C, freeze-drying for 18 h, and calcining at 600 °C for 30 min.^21^ The resulting cellular units had an average macropore size of 39.86 µm and a porosity of 90.73%. At 5 mg cm^−3^, the isotropic aerogel reached 20 mW m^−1^ K^−1^ at 25 °C, whereas a directionally frozen analogue gave approximately 26 and 35 mW m^−1^ K^−1^ along its two principal directions. Fig. 7 uses this case as a reference point: spatial regulation is useful only when it changes the continuity and orientation of the heat-transfer paths, rather than merely producing a visually heterogeneous specimen.

**Fig. 7:**
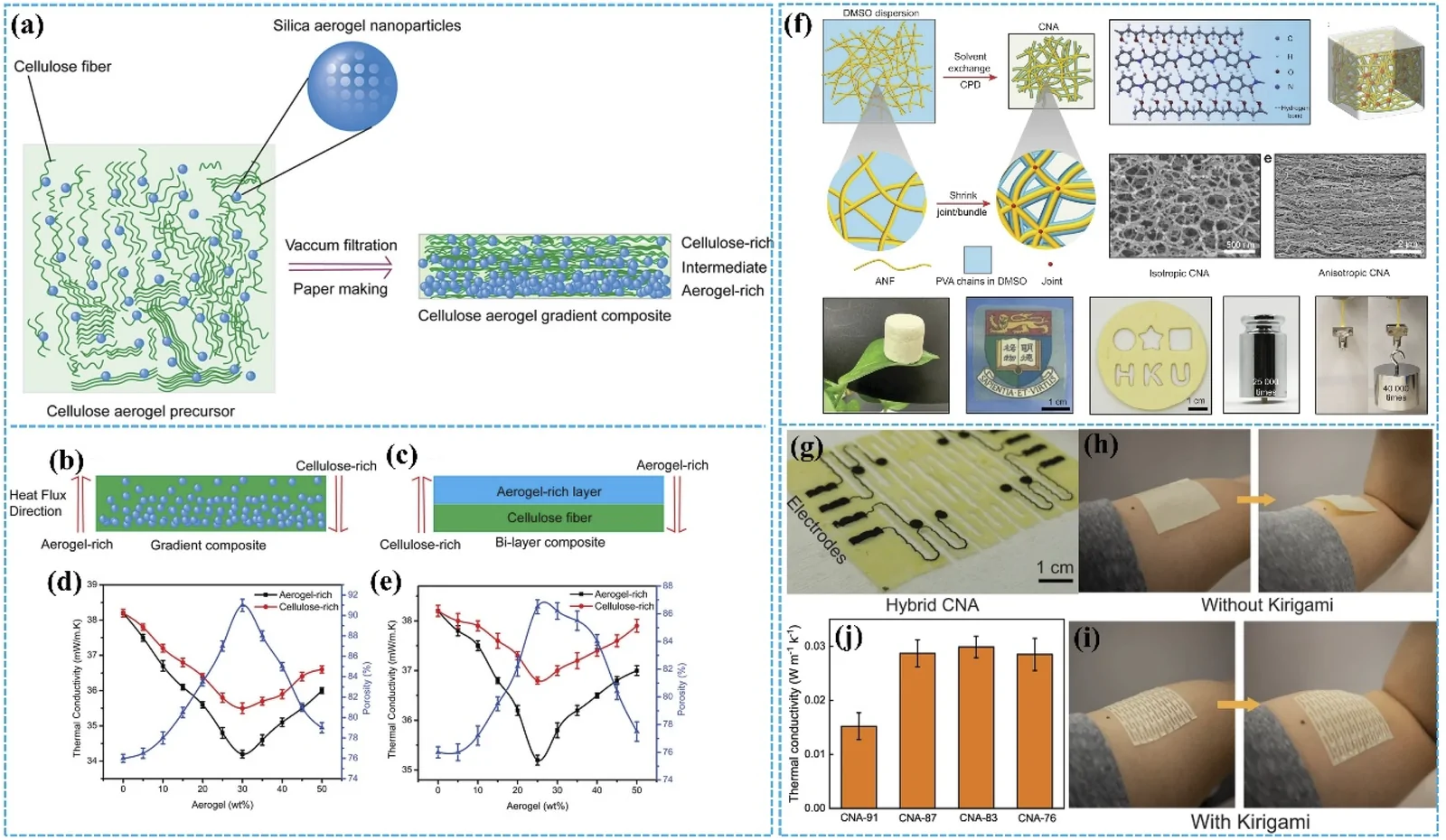
Spatial regulation of composition and junction connectivity during three-dimensional assembly. (a) Vacuum-assisted formation of a cellulose/silica-aerogel gradient containing cellulose-rich, intermediate, and aerogel-rich regions; (b and c) heat-flow orientations used to distinguish directional transport in gradient and bilayer specimens; (d) gradient-composite conductivity and porosity as the mean silica-aerogel fraction increases, showing a conductivity minimum near 30 wt% followed by an aggregation-induced increase; (e) corresponding bilayer response, with a larger difference between the two heat-flow directions because of the abrupt interface.^132^ (f) Formation mechanism, molecular interactions, isotropic and anisotropic microstructures, shapeability, and load-bearing behavior of hyperconnective ANF-PVA CNAs; (g) patterned hybrid-CNA electrode; (h and i) attachment to a curved arm without and with kirigami cuts, showing how geometric patterning improves conformability; (j) thermal conductivity of CNA-91, CNA-87, CNA-83, and CNA-76, decreasing from approximately 28 to 14 mW m^−1^ K^−1^ as porosity increases from 76% to 91%. (a–e) Reproduced with permission. Copyright 2023, Wiley-VCH GmbH.^132^ (f–j) Reproduced with permission. Copyright 2022, Springer Nature.^28^

Fig. 7 demonstrates how spatial regulation at different length scales can balance thermal insulation and mechanical integrity. In recycled-cellulose/silica composites, vacuum-assisted papermaking generated a continuous through-thickness composition gradient rather than an abrupt interface (Fig. 7a–e).^132^ Increasing the silica content initially reduced thermal conductivity by increasing porosity and disrupting cellulose-mediated heat-transfer pathways, whereas excessive silica caused aggregation and local densification. After optimization, the gradient composite achieved a thermal conductivity of 32.2 mW m^−1^ K^−1^ at a density of 0.242 g cm^−3^, together with a flexural modulus of 660 MPa. Its directional conductivity difference also confirmed that the graded phase distribution could regulate through-thickness heat transport while maintaining interfacial continuity. At a smaller scale, strongly connected aramid nanofiber/PVA networks preserved structural integrity at high porosity (Fig. 7f–j).^28^ The composite aerogel exhibited a specific tensile modulus of approximately 625.3 MPa cm^3^ g^−1^ and a fracture energy of approximately 4700 J m^−2^, while increasing porosity from 76% to 91% reduced thermal conductivity from approximately 28 to 14 mW m^−1^ K^−1^. Kirigami patterning further improved conformability on curved surfaces by releasing in-plane strain. Overall, Fig. 7 shows that composition gradients regulate heat flow at the component scale, whereas hyperconnected fibrillar junctions allow the solid fraction to be reduced without sacrificing mechanical stability.

Foaming establishes macropores before the fibrous skeleton is fixed and is governed by drainage, coalescence, and bubble-wall rupture. In foam-formed aramid/nanocellulose aerogels, cellulose-fibril morphology changes the strength and permeability of the liquid films around bubbles.^98^ Finer fibrils stabilize a larger interfacial area and subdivide the pore space, whereas poorly dispersed or excessive fibrils increase viscosity, trap nonuniform bubbles, and add solid-conduction pathways. Directly foamed inorganic nanofibrous networks provide a useful mechanistic benchmark because they demonstrate that very low density can be reached without an ice template.^133^ For polymer systems, however, the relevant optimization target is the retained bubble-size distribution after gelation and drying, not the initial foam volume.

Scaffold-assisted assembly and additive manufacturing extend structural control from pore topology to component geometry. Xue *et al.* combined direct ink writing with freezing so that nozzle path defined the macroscopic PI component while ice growth organized the internal fibrous pores.^22^ Liu *et al.* printed nanocellulose/aramid formulations around portable-electronic geometries.^134^ In both cases, shape fidelity and thermal performance originate from different variables: yield stress and shear-thinning behavior prevent filament spreading during printing, whereas fiber association, interfilament welding, and drying shrinkage determine the final density and heat-transfer path. Reporting printer resolution without final porosity, anisotropic conductivity, and interlayer strength is insufficient for comparing printed aerogels with cast monoliths.

### 3.3 Interfiber junctions and secondary networks

Assembly defines a provisional wet architecture; interfiber junctions determine whether that architecture becomes mechanically persistent. Junctions must resist stresses generated by drying, handling, compression, humidity, and thermal cycling while avoiding an excessive bonded area. This creates a nonmonotonic design problem. Too few weak contacts permit fiber sliding, pore coarsening, and thickness loss; too many broad contacts immobilize the network but increase density and solid-phase conduction. Junction quality must be assessed by retained thickness, cyclic energy loss, and conductivity after conditioning rather than by initial modulus alone.

Entanglement and hydrogen bonding preserve low solid content and permit reversible interfacial sliding, but their reversibility also makes networks vulnerable to humidity, creep, and fatigue. Zheng *et al.* addressed this problem by concentrating covalent fusion at mechanically critical cellulose contacts rather than crosslinking the entire pore wall,^14^ while Yin *et al.* crosslinked cellulose-diacetate nanofibers *in situ* and retained 99.25–99.36% porosity, a thermal conductivity of 27.2–28.4 mW m^−1^ K^−1^, a water-contact angle of 162°, and integrity through 20 000 bending cycles.^55^ Wu *et al.* used controlled reprotonation to consolidate ANF contacts.^16^ Across these chemistries, the common mechanism is spatially selective stabilization: permanent bonds prevent junction pull-out, whereas unbonded fiber segments remain available for bending and pore-volume recovery.^67^

Dual and semi-interpenetrating networks become useful when a single junction chemistry cannot provide mechanical recovery, moisture stability, and fire integrity simultaneously. Wang *et al.* combined PAI with meta-aramid fibers in a semi-interpenetrating architecture,^40^ while PI/silica and silica/aramid systems assigned mechanical continuity and thermal protection to different skeletons.^75,76,135^ The quantitative response of the aramid-fiber@silica system shows both the benefit and the limit of this strategy. Zhou *et al.* first formed a layered ANF aerogel and then deposited silica from TEOS vapor for 4–24 h.^135^ An 8-h coating increased density from 74.00 to 153.39 mg cm^−3^, but reduced conductivity from 40.22 to 30.17 mW m^−1^ K^−1^ because discontinuous, porous silica interrupted the oriented aramid heat path. At the same time, tensile strength increased from 5.80 ± 0.30 to 7.50 ± 0.50 MPa, Young's modulus increased from 49.57 ± 4.21 to 76.14 ± 5.70 MPa, the initial decomposition temperature rose from 419.2 to 467.4 °C, and the limiting oxygen index increased from 26.10 to 36.45. Extending deposition to 24 h raised the density to 298.82 mg cm^−3^ and conductivity to 54.19 mW m^−1^ K^−1^ while reducing elongation at break to 6.1%. The optimum occurred before the silica shell became a continuous thermal bridge and brittle constraint. Mineral-nanowire/ANF/cellulose and alginate/alumina networks follow the same requirement: the secondary phase should stabilize vulnerable contacts and form a fire barrier without filling the insulating pore volume.^59,61^

Localized thermal welding offers a scalable alternative for thermoplastic fibers, but only within a narrow temperature–time window. Insufficient fusion leaves contacts susceptible to shedding and creep; excessive fusion enlarges the necks into films or compact bundles. Yu *et al.* used a sea-island melt-spinning platform to control fiber fineness and contact density before assembling an aerogel felt,^20^ while PLA and polypropylene systems showed that partial welding can stabilize flexible porous bodies when only the contact points soften.^122,123^ The transferable processing variable is the ratio between welded-neck diameter and fiber diameter. This ratio should be correlated with tensile or compression fatigue and through-thickness conductivity, because a network can appear mechanically intact while progressive neck growth creates a continuous solid-conduction path.

### 3.4 Drying as structural transformation

Drying is the final stage of architecture formation rather than a passive solvent-removal operation. Freeze-drying suppresses liquid–vapor capillary pressure but introduces crystal templating, solute rejection, and sublimation gradients. Supercritical drying more faithfully preserves fine pores, yet requires solvent exchange, pressure equipment, and batch operation. Ambient drying is simpler, but the capillary pressure generated by an evaporating meniscus scales inversely with pore radius and can collapse the finest pores first. Drying routes must consequently be compared using linear and volumetric shrinkage, density gradients, surface area, and post-drying mechanical and thermal performance, together with cycle time and solvent recovery.^68,136,137^

Freeze-drying remains common because the frozen solvent temporarily supports fragile networks. Its principal limitation is that the same support phase becomes a structural template. In thick components, spatial differences in nucleation, crystal growth, and sublimation create density gradients that are not evident in small laboratory specimens. Li *et al.* overcame the alignment imposed by columnar ice through the crushed-ice/recasting strategy described above;^21^ the large samples retained compression behavior and thermal conductivity comparable to small specimens. Li *et al.* coordinated solvent recovery and multiscale PI processing to demonstrate recyclable aerogel manufacture.^24^ The scale-up question is not whether a larger frozen gel can be dried, but whether the freezing history and vapor-removal path remain sufficiently uniform to reproduce the designed architecture.

Ambient-pressure routes become credible when capillary stress is reduced below the strength of the wet skeleton or redistributed through a hierarchy of load-bearing elements. Cheng *et al.* combined Ca^2+^ crosslinking with low-surface-tension solvent exchange before rapidly air-drying wet-spun ANF fibers.^138^ This coupled treatment limited shrinkage to 29.0%, retained a specific surface area of 225.0 m^2^ g^−1^, and produced fibers with a tensile strength of 13.5 MPa and a toughness of 7.0 MJ m^−3^. Woven textiles exhibited a conductivity of approximately 38.5 mW m^−1^ K^−1^ and remained effective from −196 to 400 °C. The result identifies the two functions that must be separated during ambient drying: crosslinking raises the allowable capillary stress, whereas solvent exchange lowers the applied capillary stress. Fig. 8 extends this principle from individual aerogel fibers to molded foams, fiber-reinforced composites, and hyperconnected silicone networks.

**Fig. 8:**
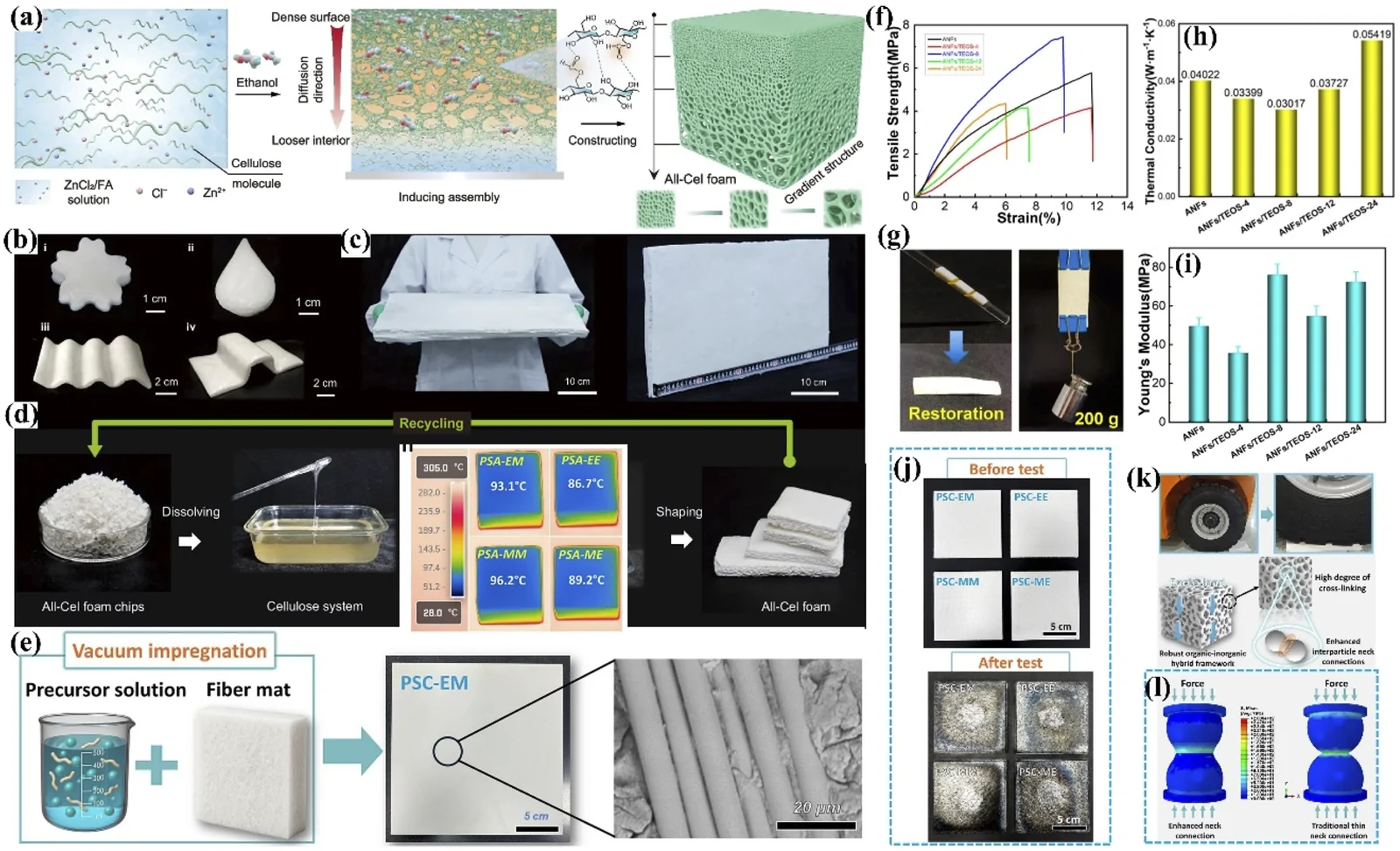
Drying-compatible stabilization from molecular assembly to reinforced networks. (a) Ethanol-induced assembly of cellulose chains and development of a honeycomb-like gradient All-Cel foam; (b) direct molding and water-assisted reshaping before ethanol exchange; (c) 47 × 28 × 3 cm panel demonstrating scalable ambient processing; (d) closed-loop reconstruction through shredding, redissolution, remolding, and renewed ethanol-induced assembly. (e) Vacuum impregnation of a quartz-fiber mat with a silicone-aerogel precursor and the resulting PSC-EM macro/microstructure. (f) Tensile stress–strain curves of ANF and ANF/TEOS aerogels after different TEOS-vapor deposition times; (g) foldability, elastic restoration, and support of a 200-g load; (h) conductivity decreasing from 40.22 to 30.17 mW m^−1^ K^−1^ after 8 h deposition, then rising to 54.19 mW m^−1^ K^−1^ after 24 h as the silica shell becomes dense and continuous; (i) Young's modulus, with the 8-h sample reaching 76.14 ± 5.70 MPa. (j) Quartz-fiber/silicone-aerogel composites before and after severe thermal exposure; (k) 5-ton-forklift test and multiscale thick-neck reinforcement mechanism; (l) finite-element comparison showing stress localization at a thin particle neck and broader load distribution after neck enhancement. (a–d) Reproduced with permission. Copyright 2026, Springer Nature.^139^ (e and j–l) Reproduced with permission. Copyright 2026, Wiley-VCH GmbH.^140^ (f–i) Reproduced with permission. Copyright 2022, MDPI AG.^135^

Fig. 8 summarizes three approaches for strengthening porous networks while reducing damage during drying and subsequent loading. In the all-cellulose foam, ethanol acted simultaneously as a nonsolvent and an assembly trigger, inducing chain association and generating a honeycomb-like pore gradient without freeze-drying (Fig. 8a–d).^139^ This method produced a recyclable panel as large as 47 × 28 × 3 cm, with a density of 0.12 ± 0.01 g cm^−3^, a compressive modulus of 11.8 ± 1.3 MPa, and a thermal conductivity of 47–62 mW m^−1^ K^−1^. In the aramid nanofiber aerogel, controlled TEOS vapor deposition reinforced fiber junctions with a porous silica coating (Fig. 8f–i).^135^ An 8 h treatment increased tensile strength from 5.80 ± 0.30 to 7.50 ± 0.50 MPa and reduced thermal conductivity from 40.22 to 30.17 mW m^−1^ K^−1^; however, extending deposition to 24 h increased conductivity to 54.19 mW m^−1^ K^−1^ because the thicker silica layer raised density and created continuous thermal bridges. The quartz-fiber/silicone composite employed a complementary multiscale strategy in which continuous fibers transferred long-range loads, while hyperbranched siloxane nodes enlarged interparticle necks and dispersed local stress (Fig. 8e and j–l).^140^ The optimized silicone aerogel exhibited a compressive strength of 7.1 MPa and a conductivity of 32 mW m^−1^ K^−1^, while the quartz-fiber composite reached a tensile strength of 28.1 MPa and retained structural integrity under severe mechanical and thermal exposure. Fig. 8 shows that chain entanglement, controlled interfacial coatings, and reinforced junctions can improve drying compatibility and mechanical stability, but excessive solid continuity must be avoided because it increases thermal conduction.

## 4 Structure-mediated regulation of thermal transport

The effective thermal conductivity of a porous fibrous aerogel can be expressed as the combined contributions of solid conduction, gas conduction, thermal radiation, residual convection, and moisture-mediated transport. These terms do not vary in parallel. Increasing density improves skeletal continuity and suppresses large-scale gas motion, but also raises solid conduction; adding an infrared opacifier reduces radiative transfer at high temperature, but may create new conductive contacts; and crosslinking preserves pore geometry while enlarging junction area. Structural regulation must be evaluated from the net conductivity and from the persistence of the architecture after compression, humidity exposure, and thermal cycling, rather than from porosity or polymer identity alone.

Radiative-transfer measurements, hygrothermal testing, mixed-fiber experiments, and fabric-scale modeling provide the basis for this integrated view.^10–12,25^ Throughout this review, initial thermal conductivity denotes the value measured for an as-prepared specimen under a specified baseline condition, whereas retained thermal insulation refers to conductivity, thermal resistance, or heat-shielding performance measured after a defined mechanical, hygrothermal, thermal, or fire exposure. The distinction is essential because a structure that minimizes transport initially may lose that advantage after shrinkage, pore collapse, moisture uptake, or interfacial damage. Conductivity values are interpreted together with pressure, humidity, heat-flow direction, thickness, uncertainty, and exposure history. The following sections organize structural strategies by their primary heat-transfer pathway and, where evidence is available, explicitly separate the initial transport benefit from the conditions under which that benefit is retained.

### 4.1 Fiber and junction design for limiting solid conduction

Solid heat is transported through fibers and their contacts. Finer, high-aspect-ratio fibers can percolate at low solids content, reduce the load-bearing cross-section, and introduce more phonon-scattering interfaces. The benefit is not monotonic: excessive refinement promotes bundling and drying-induced buckling, whereas too many welded contacts shorten the thermal path. Fiber diameter must be interpreted together with curvature, bundle fraction, node spacing, and contact area.

Quantitative results show that curvature and multiscale entanglement are at least as important as nominal fiber diameter. Curly PI nanofibers produced an aerogel with a density of 6.8 mg cm^−3^, a thermal conductivity of 24.5 mW m^−1^ K^−1^, and nearly complete recovery after 1500 deformation cycles.^18^ Biomimetic helical micro/nanofiber sponges reached 7.1 mg cm^−3^ and 99.6% porosity, sustained 200% tensile strain and 1000 tensile/compression cycles, and retained a conductivity of 24.85 mW m^−1^ K^−1^ after repeated washing.^70^ In the loofah-inspired system, a rigid-microfiber/flexible-nanofiber network achieved 3 mg cm^−3^, 99.7% porosity, 25.3 mW m^−1^ K^−1^, and survived 1000 compression cycles.^141^ A low solid fraction becomes mechanically usable only when curved fibers and hierarchical entanglements redistribute strain without forming dense bundles.

Junction topology creates a second optimization. In an ANF/PVA aerogel, an average nodal connectivity of about 5.4 and welded contacts yielded a tensile strength of 6.3 MPa at 76% porosity and a fracture energy of 4697.6 J m^−2^; increasing porosity to 91% reduced thermal conductivity from approximately 28 to 14 mW m^−1^ K^−1^.^28^ The data demonstrate that connectivity can raise mechanical efficiency by orders of magnitude without uniformly increasing solid content, but also expose a trade-off between the lowest conductivity and maximum strength. Selective covalent fusion provides a related solution: localized permanent nodes preserve long, low-density spans instead of coating the entire skeleton.^14^ Thus, the relevant variable is the number and geometry of mechanically effective contacts, not crosslinker concentration alone.

A practical junction design should report coordination number, contact dimensions, and the fraction of reversible and permanent bonds together with conductivity before and after cycling. Added contacts are thermally justified only when the structural collapse and conductivity increase that they prevent during service exceed their initial solid-conduction penalty.

### 4.2 Pore architecture for suppressing gas conduction and convection

Gas conduction decreases when characteristic pore dimensions approach the mean free path of gas molecules, because gas-wall collisions interrupt molecular energy transport. Nanofibers are effective in this regime because they create small pore throats and high interfacial area without forming a continuous dense wall. However, the arithmetic mean pore diameter is insufficient: a small fraction of connected cracks or macropores can dominate gas transport and permit buoyancy-driven circulation.

Hierarchical pores assign different transport and mechanical functions to different length scales: nanoscale throats increase gas-wall collisions, intermediate pores accommodate fiber bending, and controlled macropores distribute stress or shorten solvent-removal paths without becoming continuous through-thickness channels. Cellular PI and ANF networks, nacre-like cellulose layers, and plant-cell-wall-inspired composites illustrate this division of roles.^23,34,95,142^ Recent nanocellulose architectures make the relationship more explicit. A biomimetic tracheal TOCNF/PVTMS/LDH aerogel combined directionally formed tubular pores with localized inorganic/organosilicon reinforcement, reaching a radial thermal conductivity of 26.5 mW m^−1^ K^−1^ while preserving a compressive modulus of 102.8 kPa and UL-94 V-0 flame retardancy.^82^ Sequential pre-freezing has also produced Janus CNF/PET cryogels with porosity above 96% and independently tunable pore gradients.^83^ Although that Janus system was developed for filtration, rather than insulation, it demonstrates that pore-size distribution and through-thickness connectivity can be programmed independently of total porosity. For thermal design, the transferable descriptors are pore-throat distribution, connected macropore fraction, permeability, and the stability of these quantities after drying and compression.

The same requirement becomes more stringent as the operating temperature rises. As summarized in Fig. 9a, b and g–n, turbulent-flow-assisted centrifugal spinning produced large, shapeable medium-entropy (La_0.5_Y_0.5_)_2_Zr_2_O_7_ fibrous aerogels whose grains remained approximately 405.8 nm after prolonged ultrahigh-temperature exposure, with volume shrinkage below 3%, a strength increase exceeding 26%, and a thermal conductivity of 101.1 mW m^−1^ K^−1^ at 1273 K.^143^ The value is higher than room-temperature polymer-aerogel records, but the retained pore architecture under this radiative and high-temperature regime is the relevant performance criterion. Pore hierarchy is beneficial only while large pores remain disconnected across the heat-flow direction and fine throats survive thermal or mechanical evolution. Accordingly, pore-size distributions should be reported together with three-dimensional connectivity, permeability, shrinkage, and conductivity before and after exposure. Directional conductivity and fiber alignment, including the SiC@SiO_2_ example in Fig. 9c–f, are treated separately in Section 4.3 to distinguish pore-size effects from anisotropic transport.

**Fig. 9:**
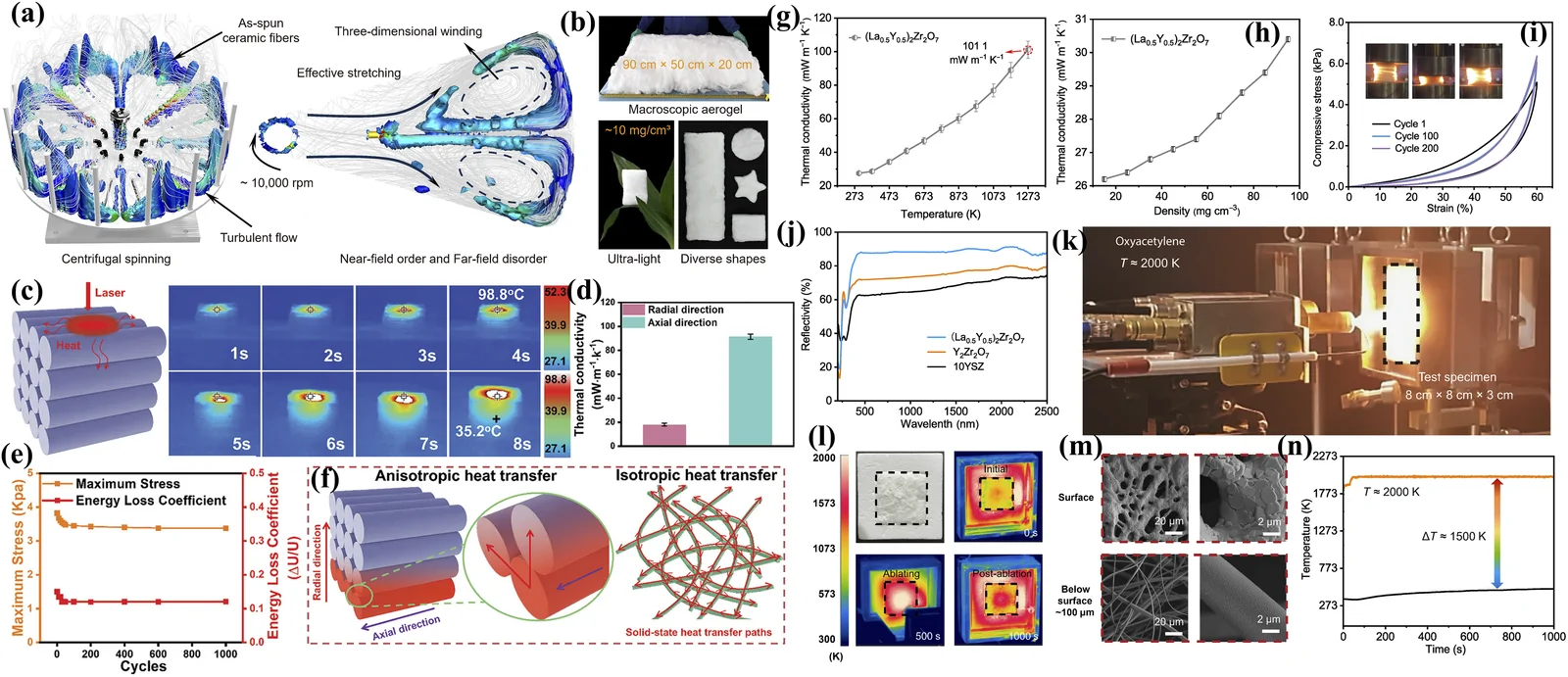
Coupling pore hierarchy, orientation, and high-temperature stability. (a and b) Turbulent-flow-assisted centrifugal spinning and macroscopic ZYL aerogels with scalable output, low density, and shape versatility. (c–f) Laser-heating experiment, axial/radial thermal conductivity, cyclic energy loss and maximum stress, and heat-transfer mechanism of oriented A-SiC@SiO_2_ fibrous aerogels. (g and h) Temperature- and density-dependent conductivity and spectral reflectivity of ZYL aerogels; (i) compression at 60% strain for 200 cycles at 1500 K; (j–n) oxyacetylene-flame test, transient infrared images, microstructure before/after ablation, and the resulting approximately 1500 K temperature difference across the specimen. (a and b) Reproduced with permission. Copyright 2026, American Association for the Advancement of Science.^143^ (c–f) Reproduced with permission. Copyright 2025, Wiley-VCH GmbH.^144^ (g–n) Reproduced with permission. Copyright 2026, American Association for the Advancement of Science.^143^

### 4.3 Anisotropic and gradient architectures for directional heat-flow control

Orientation converts thermal conductivity from a scalar screening value into a directional transport property. Continuous aromatic chains and ceramic fibers generally conduct more efficiently along their axes than across fiber–fiber or fiber–matrix interfaces; accordingly, alignment can either suppress or accelerate heat flow depending on its relation to the thermal gradient. The oriented SiC@SiO_2_ aerogel in Fig. 9 provides a direct benchmark, with transverse and axial conductivities of 0.018 and 0.0914 W m^−1^ K^−1^, respectively, corresponding to an anisotropy factor of 5.08.^144^ A recent PVTMS-modified TEMPO-oxidized cellulose nanofiber aerogel exhibited an even larger directional contrast, with reported axial and radial conductivities of 11.5 and 115.2 mW m^−1^ K^−1^, respectively.^81^ These data show why a single conductivity value is insufficient for aligned networks. Bio-derived architectures reinforce the same principle at larger length scales: silicon-modified loofah carbon aerogels retained vascular channels that localized solar heat while simultaneously sustaining directional mass transport during interfacial evaporation.^84^ Because the latter framework is carbonized, it is used here as a transport-architecture benchmark rather than classified as a polymer fibrous aerogel. Across these systems, orientation factor, principal-direction conductivity, and their retention after cycling are the minimum descriptors needed to establish a directional thermal advantage.

Gradients become advantageous when opposite faces experience different thermal, mechanical, or environmental boundary conditions. Directionally frozen PI lamellae can be oriented to retain load-bearing continuity while limiting through-thickness heat flow;^33^ composition-gradient PI and cellulose architectures place the more stable phase toward the hot boundary and the more compliant phase toward the cold boundary;^19,132^ and fluorinated PI/paraffin sandwiches spatially separate steady thermal resistance from transient latent-heat buffering.^118^ Janus pore architectures extend this concept by assigning different pore-size distributions to adjacent layers: sequentially pre-frozen CNF/PET cryogels maintained porosity above 96% while creating mechanically robust layers with distinct filtration functions.^83^ This study does not provide direct thermal-conductivity evidence, but it demonstrates an experimentally accessible route for decoupling pore gradient from overall solid fraction. For thermal applications, such gradients should be validated by through-thickness and in-plane conductivity, interfacial resistance, and retained architecture after thermal or moisture cycling. Spatial separation is useful only when the interface between functional zones remains mechanically stable and does not itself become a low-resistance heat bridge.

Layered interfaces add thermal resistance and deflect cracks only if adjacent layers remain mechanically compatible. Multilayer PI, aramid composites, PI/silica, and silica-fiber/SiC networks separate load bearing, radiative attenuation, and fire protection.^35,96,145,146^ Their validation should include axial and transverse conductivity, interlaminar strength, directional shrinkage, and thermal-cycle durability; otherwise an apparent thermal barrier may simply be an incipient delamination defect.

### 4.4 Hybrid networks for radiative heat management and fire protection

Four terms are distinguished in this section because they describe different responses and require different evidence. Flame retardancy refers to resistance to ignition, flame spread, and heat/smoke release and is commonly evaluated by LOI, UL-94, and cone calorimetry. Fire resistance describes the ability of a material or component to preserve structural integrity or a barrier function during sustained fire exposure and must be reported together with the imposed heat source, duration, and failure criterion. Thermal shielding denotes attenuation of transmitted heat, quantified by back-face temperature, heat flux, or thermal resistance, and can occur even when a material is not intrinsically flame-retardant. Ablation resistance concerns mass loss, surface recession, and residue stability under intense heat flux and is quantified by mass or linear ablation rates together with post-exposure morphology. These metrics are complementary but not interchangeable; a direct-flame photograph alone cannot establish flame retardancy, fire resistance, thermal shielding, and ablation resistance simultaneously.

Radiation becomes increasingly important at elevated temperature and in optically thin networks. Fig. 10 compares three ways to introduce a secondary phase without uniformly filling the pores. TiO_2_ particles in a silica nanofibrous aerogel reduced room-temperature conductivity to 0.019 W m^−1^ K^−1^, limited infrared transmittance to approximately 67% at 3 µm, and retained a compressive strength of 3.22 kPa; under the same flame-heating condition, the TiO_2_-containing sample showed a surface temperature of 43.3 °C *versus* 57.7 °C for the unfilled nanofibrous aerogel (Fig. 10b and d).^147^ In ANF@Al_2_O_3_ aerogels, Al_2_O_3_ decreased thermal conductivity from 34.1 to 30.0 mW m^−1^ K^−1^ while the optimized modulus reached 41.869 MPa, 2.1 times that of the unmodified ANF network (Fig. 10e).^148^ An opacifier or ceramic coating is effective when it simultaneously interrupts radiation and reinforces existing pore walls rather than forming a separate percolating solid network.^149–152^

**Fig. 10:**
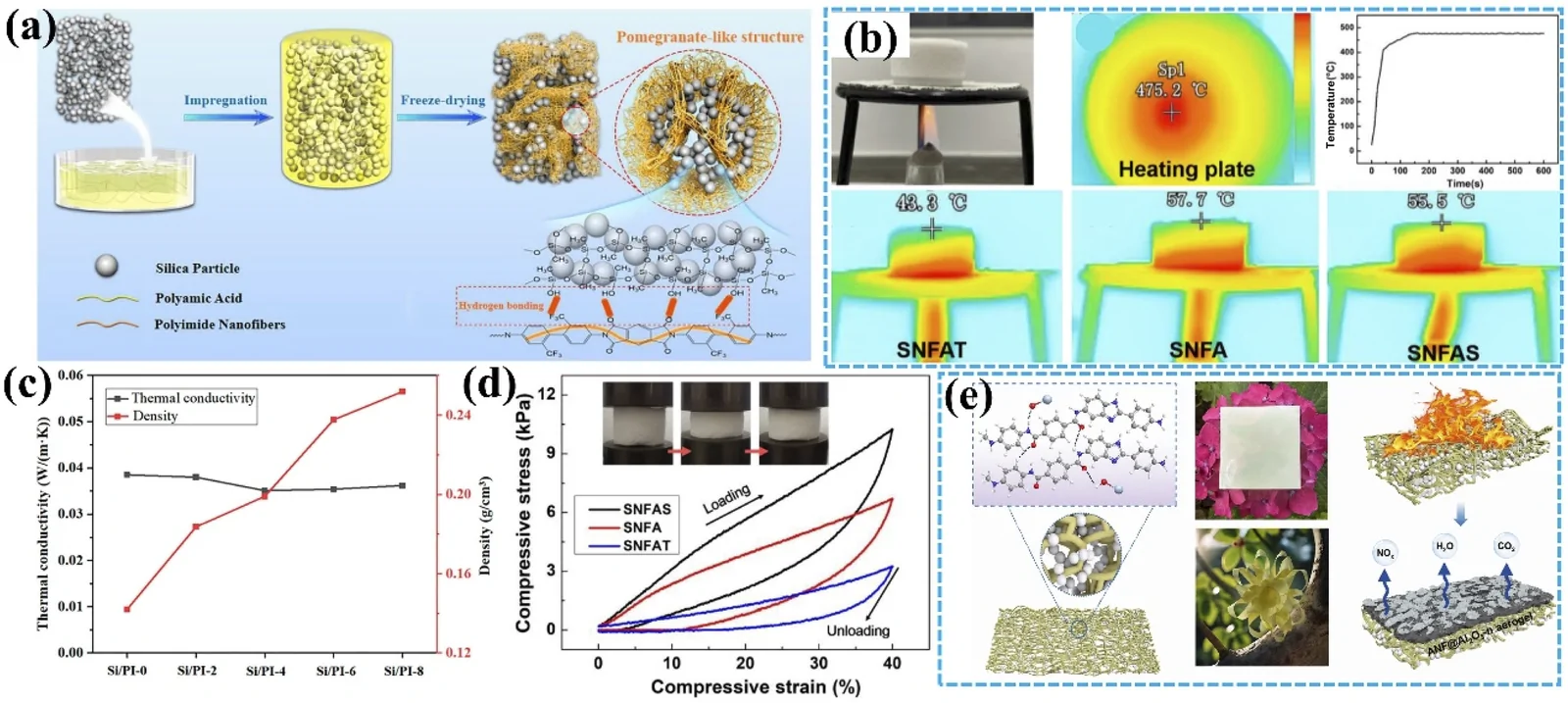
Localized hybrid phases for thermal and fire protection. (a) Formation of a pomegranate-like Si/PI network in which PI nanofibers interlock silica particles; (c) density and thermal conductivity as PI content changes. (b and d) Direct-flame infrared images and 40%-strain compression curves of TiO_2_-filled (SNFAT), unfilled (SNFA), and SiO_2_-particle-filled (SNFAS) silica nanofibrous aerogels. (e) Slit-pore ANF@Al_2_O_3_ aerogel integrating optical transmission, thermal insulation, and an Al_2_O_3_-supported fire barrier. (a and c) Reproduced with permission. Copyright 2025, MDPI AG.^145^ (b and d) Reproduced with permission. Copyright 2022, MDPI AG.^147^ (e) Reproduced with permission. Copyright 2025, Springer Nature.^148^

The same additives become unfavorable when they agglomerate or percolate. CNT/ANF, PI/MXene, MXene/aramid/PI, PI/graphene, and all-carbon networks add sensing, electromagnetic response, or active heating, but they also increase solid contact continuity.^9,105,153,154^ These systems should report temperature-dependent passive conductivity and the unpowered temperature field. A conductive phase is most useful when confined to a thin skin or an isolated pathway that does not bridge the protected faces.

Fire resistance requires preservation of a coherent barrier after polymer softening, decomposition, or charring, whereas flame retardancy additionally requires evidence that ignition, flame propagation, and heat/smoke release are suppressed. The pomegranate-like Si/PI network in Fig. 10a and c illustrates the barrier route: PI nanofibers interlocked silica particles, giving an optimized compressive strength of 2.86 MPa while maintaining a thermal conductivity of 0.035 W m^−1^ K^−1^, and the composite remained intact during a 1300 °C flame exposure for 20 min.^145^ PBO occupies a distinct regime because its rigid conjugated heteroaromatic backbone provides high intrinsic thermal and flame stability before a secondary flame-retardant phase is introduced. Protonation-enabled, hydrogen-bond-assisted PBO aerogels achieved UL-94 V-0 and an LOI of 45–52%, together with a peak heat-release rate of 23.8 kW m^−2^, total heat release of 2.3 MJ m^−2^, and total smoke release of 3.3 m^2^ m^−2^.^101^ PNF/nanodiamond composite papers reached UL-94 V-0 and LOI values of 56–63%, further supporting the intrinsic flame-resistant character of PBO-rich networks, although these dense papers should not be treated as direct aerogel-insulation evidence.^155^ Together with mineral- and char-forming cellulose and alginate systems,^59–62,102,156–162^ these results indicate that an effective fire-protective architecture must form a continuous protective residue during exposure while avoiding continuous dense additive pathways that would increase solid conduction during normal service.

Accordingly, hybridization should be treated as an architecture rather than a formulation. Ceramic-rich hot faces, sparse opacifiers on pore walls, and sol–gel bridges at selected nodes can suppress radiation and preserve residue integrity with a smaller penalty in density and compliance than uniform bulk loading.

### 4.5 Retaining thermal insulation under moisture and environmental exposure

Moisture replaces low-conductivity air, forms liquid bridges, and can reorganize hydrophilic junctions. A PVDF/PI nanofiber composite combined a 152° water contact angle with a conductivity of 31.0 mW m^−1^ K^−1^ at ambient conditions; even at 80 °C and 100% relative humidity, conductivity remained 48.6 mW m^−1^ K^−1^.^113^ A PLA hollow-fiber aerogel reached 23.03 mg cm^−3^, 91% porosity, a 154.3° contact angle, and approximately 0.029 W m^−1^ K^−1^; it retained 84.92% of its initial compressive stress after 100 cycles at 60% strain.^122^ The comparison shows that low surface energy must be coupled to a fatigue-resistant hollow or fibrous skeleton: contact angle alone does not reveal whether humid air penetrates the interior or whether the coating cracks during use.

Environmental reliability should be quantified by vapor-sorption isotherms, wet-state conductivity, swelling and drying hysteresis, permeability, and retained coating integrity after compression and wet–dry or freeze–thaw cycling.^11^ The decisive metric is retained thermal resistance after environmental exposure, not the initial droplet contact angle.

Table 2 consolidates these relationships using representative fibrous aerogel systems for which structural descriptors and performance were reported together. Conductivities below 30 mW m^−1^ K^−1^ were obtained through different routes, including fiber curvature and multiscale entanglement,^18,70^ localized high-connectivity junctions,^28^ directional pore alignment,^144^ and controlled hybridization.^147,148^ The accompanying data also show why conductivity alone is insufficient: the oriented SiC@SiO_2_ aerogel exhibited a 5.08-fold directional difference,^144^ the ANF/PVA network traded the lowest conductivity at 91% porosity against maximum strength at 76% porosity,^28^ and the conductivity of the PVDF/PI network increased from 31.0 to 48.6 mW m^−1^ K^−1^ at 80 °C and 100% relative humidity.^113^ Accordingly, Table 2 links each structural gain to its principal failure risk and to the minimum measurements needed for a meaningful comparison.

**Table 2:** Structural variable–heat-transfer pathway–beneficial regime relationships, overextension risks, and validation requirements in representative fibrous aerogels

Structural variable	Representative system and measurable descriptor	Affected heat-transfer pathway and quantitative evidence	Beneficial regime and overextension risk	Validation for retained performance	Ref.
Curved ultrafine fibers	Directly synthesized curly PI nanofiber network	Primary pathway: solid conduction and retention of the pore network. Density: 6.8 mg cm^−3^; thermal conductivity: 24.5 mW m^−1^ K^−1^; nearly complete recovery after 1500 deformation cycles	Curvature redistributes strain while maintaining long thermal paths. Excess refinement can promote bundles, drying-induced buckling, and local densification	Fiber-diameter and curvature distributions; bundle fraction; shrinkage; conductivity before/after cycling	18
Helical multiscale entanglement	Biomimetic helical micro/nanofiber sponge	Primary pathway: solid conduction and gas transport through mechanically retained pores. Density: 7.1 mg cm^−3^; porosity: 99.6%; conductivity: 24.85 mW m^−1^ K^−1^ after repeated washing; 200% tensile strain and 1000 tensile/compression cycles	Helical fibers provide extensibility without a dense supporting phase. Overly open macropores or fiber bundles can raise gas transport and spatial variability	Pore-throat distribution; tensile/compressive recovery; wash durability; wet/dry conductivity	70
Localized high-connectivity junctions	Welded ANF/PVA network with average nodal connectivity of approximately 5.4	Primary pathway: solid conduction across fiber junctions. At 76% porosity: tensile strength 6.3 MPa and fracture energy 4697.6 J m^−2^; increasing porosity to 91% reduced conductivity from approximately 28 to 14 mW m^−1^ K^−1^	Mechanically effective nodes improve strength, but maximum strength and minimum conductivity occur at different solid fractions. Continuous welding would shorten the thermal path	Coordination number; contact area; porosity; fracture energy; conductivity–density relation; cyclic recovery	28
Collapse-resistant hierarchical pores	Ambient-dried plant-cell-wall-inspired cellulose composite	Primary pathway: gas conduction/convection and collapse-induced solid conduction. Drying shrinkage: 8.56%; thermal conductivity: 0.0381 W m^−1^ K^−1^	A reinforced multiscale skeleton prevents capillary collapse, although excessive reinforcement increases solid conduction and wall densification	Shrinkage and density before/after drying; 3D pore connectivity; throat-size distribution; pressure-dependent conductivity	23
Directional pore and fiber alignment	Oriented SiC@SiO_2_ fibrous aerogel used as a high-temperature architecture benchmark	Primary pathway: direction-dependent solid and gas conduction. Transverse conductivity: 0.018 W m^−1^ K^−1^; axial conductivity: 0.0914 W m^−1^ K^−1^; anisotropy factor: 5.08	Alignment perpendicular to heat flow increases interfacial resistance, whereas axial alignment creates a thermal bridge. Direction must be specified	Axial/transverse conductivity; orientation factor; directional mechanics and shrinkage; thermal cycling	144
Dispersed infrared opacifier	TiO_2_-filled silica nanofibrous aerogel (SNFAT)	Primary pathway: radiative heat transfer. Conductivity: 0.019 W m^−1^ K^−1^; infrared transmittance approximately 67% at 3 µm; compressive strength: 3.22 kPa; flame-side surface temperature: 43.3 °C *versus* 57.7 °C for unfilled SNFA	Dispersed particles suppress radiation, but agglomeration or percolation increases density, solid contacts, and local defects	Particle loading/distribution; spectral response; temperature-dependent conductivity; density; flame-test boundary conditions	151
Interlocked polymer-inorganic fire barrier	Pomegranate-like Si/PI network with PI nanofibers interlocking silica particles	Primary pathway: radiative transfer and fire-induced heat/mass transport. Compressive strength: 2.86 MPa (approximately 12-fold that of unmodified silica aerogel); conductivity: 0.035 W m^−1^ K^−1^; 1300 °C flame resistance for 20 min; water contact angle: 123°	Interlocking improves residue integrity and load transfer. Uniform pore filling would raise density, embrittlement, and solid conduction	Filler continuity; residue morphology; high-temperature conductivity; mass/shrinkage after flame; compressive retention	149
Conformal ceramic reinforcement	Slit-pore ANF@Al_2_O_3_ composite aerogel	Primary pathway: radiative transfer coupled with solid conduction. Al_2_O_3_ reduced conductivity from 34.1 to 30.0 mW m^−1^ K^−1^; optimized modulus reached 41.869 MPa, 2.1 times that of the unmodified ANF network	A thin ceramic phase can reinforce existing pore walls while limiting radiative transfer. A continuous thick coating would enlarge conductive contacts	Coating thickness/coverage; modulus; pore retention; optical response; temperature-dependent conductivity	152
Hydrophobic co-network	PVDF/PI nanofiber composite with low-surface-energy protection	Primary pathway: moisture-mediated transport and gas conduction. Water contact angle: 152°; conductivity: 31.0 mW m^−1^ K^−1^ at ambient conditions and 48.6 mW m^−1^ K^−1^ at 80 °C/100% RH	Hydrophobicity delays liquid penetration but does not eliminate vapor uptake or temperature-driven gas conduction. Coating defects can dominate long-term behavior	Vapor sorption; wet-state conductivity; permeability; wet-dry cycling; coating-crack and adhesion assessment	118
Hydrophobic hollow-fiber skeleton	Flexible superhydrophobic PLA hollow-fiber aerogel	Primary pathway: moisture-mediated transport and gas conduction. Density: 23.03 mg cm^−3^; porosity: 91%; water contact angle: 154.3°; conductivity: approximately 0.029 W m^−1^ K^−1^; 84.92% stress retention after 100 cycles at 60% strain	Hollow fibers couple moisture resistance with recoverability, but wall collapse, creep, or coating damage can create conductive bridges during service	Hollow-wall integrity; creep; cyclic stress retention; humidity-conditioned conductivity; coating retention	127

## 5 Thermal performance and practical applications

The preceding structural strategies become meaningful only when their thermal benefits persist under application-specific boundary conditions. Clothing, buildings, batteries, transportation, and aerospace systems share the need to restrict heat transfer but differ in thickness, mass, heat flux, pressure, humidity, deformation, fire exposure, and qualification time. This section moves from record values to operating envelopes and examines which architectures, tests, and manufacturing constraints determine practical viability.

### 5.1 Evaluation of thermal insulation performance

The practical value of a fibrous aerogel cannot be inferred from its minimum initial thermal conductivity. Initial conductivity should be reported only as a baseline property of the as-prepared structure under defined density, thickness, pore gas, pressure, mean temperature, heat-flow direction, humidity, contact pressure, and test method. Retained thermal insulation, by contrast, quantifies how much of that baseline resistance survives a defined mechanical or environmental history. Thin transient-tested membranes, guarded-hot-plate monoliths, and compressed felts are not directly comparable, and anisotropic or gradient materials require measurements along both principal directions with the specimen orientation relative to the heat source explicitly stated.

Thermal performance should consequently be reported as an operating envelope that contains both initial and retained values rather than as a single record point. The CPI fabric discussed below, for example, increases from 24.2 mW m^−1^ K^−1^ at −50 °C to 70.2 mW m^−1^ K^−1^ at 150 °C.^163^ This approximately threefold change is intrinsic to the tested temperature window and illustrates why room-temperature conductivity cannot be used as a service limit. The same dataset should include shrinkage, thickness retention, recovery at a defined strain and cycle count, and conductivity after humidity, mechanical, or thermal aging so that a reduction in insulation can be assigned to a measurable structural change rather than to an unspecified conditioning history.

Representative values also show why polymer families cannot be ranked in isolation. Ultralight polymeric networks can reach 14–30 mW m^−1^ K^−1^ near room temperature,^18,28,70^ whereas oxide fibrous aerogels may remain below approximately 0.10 W m^−1^ K^−1^ above 1000 °C.^144,143,164^ The latter values are numerically higher but correspond to a much harsher radiative and oxidative regime. Comparisons must match temperature, pressure, humidity, density, thickness, and service function rather than polymer name.

Mechanical data should likewise reflect retention of insulation. A lamellar ZrO_2_–SiO_2_ nanofibrous aerogel recovered from 90% strain, delivered 950 kPa at 90% compression, showed zero permanent deformation after 1000 cycles, and maintained a thermal conductivity of 0.0268 W m^−1^ K^−1^.^161^ This combination is more informative than a single peak stress because it demonstrates that both the pore geometry and the associated resistance to heat flow are retained after repeated loading.

### 5.2 Wearable thermal management

Wearable insulation places unusually strict constraints on mass, thickness, flexibility, breathability, abrasion, washability, and moisture comfort. A bulk monolith with excellent conductivity but poor bending or sewing tolerance is unsuitable. Conversely, an open textile that is comfortable and flexible may permit excessive air exchange. Three-dimensional fibrous aerogels can function as inserts, felts, liners, or cellular membranes when their pore network is sufficiently fine to restrict gas transport and sufficiently resilient to survive body motion.

Wearable insulation must combine low areal mass with softness, breathability, bending recovery, moisture comfort, and wash durability. Fig. 11a–e illustrates how radial architecture controls these coupled properties. Bio-inspired cellular ANF fibers used a dense shell and cellular core to reach 74.6 MPa tensile strength while keeping bending and compression stresses as low as 33.8 and 39.8 kPa, respectively; their conductivity was 28.4–32.1 mW m^−1^ K^−1^ and increased only from 28.4 to 29.7 mW m^−1^ K^−1^ as relative humidity rose from 30% to 90%.^165^ Gradient all-nanostructured aramid fibers further combined 150 nm sheath pores with 600 nm core pores, producing a radial conductivity of 0.0228 W m^−1^ K^−1^, a tensile strength of 29.5 MPa, and 39.2% fracture strain (Fig. 11b–e).^166^ The data show that radial gradients can raise interfacial thermal resistance without sacrificing the continuity required for weaving.

**Fig. 11:**
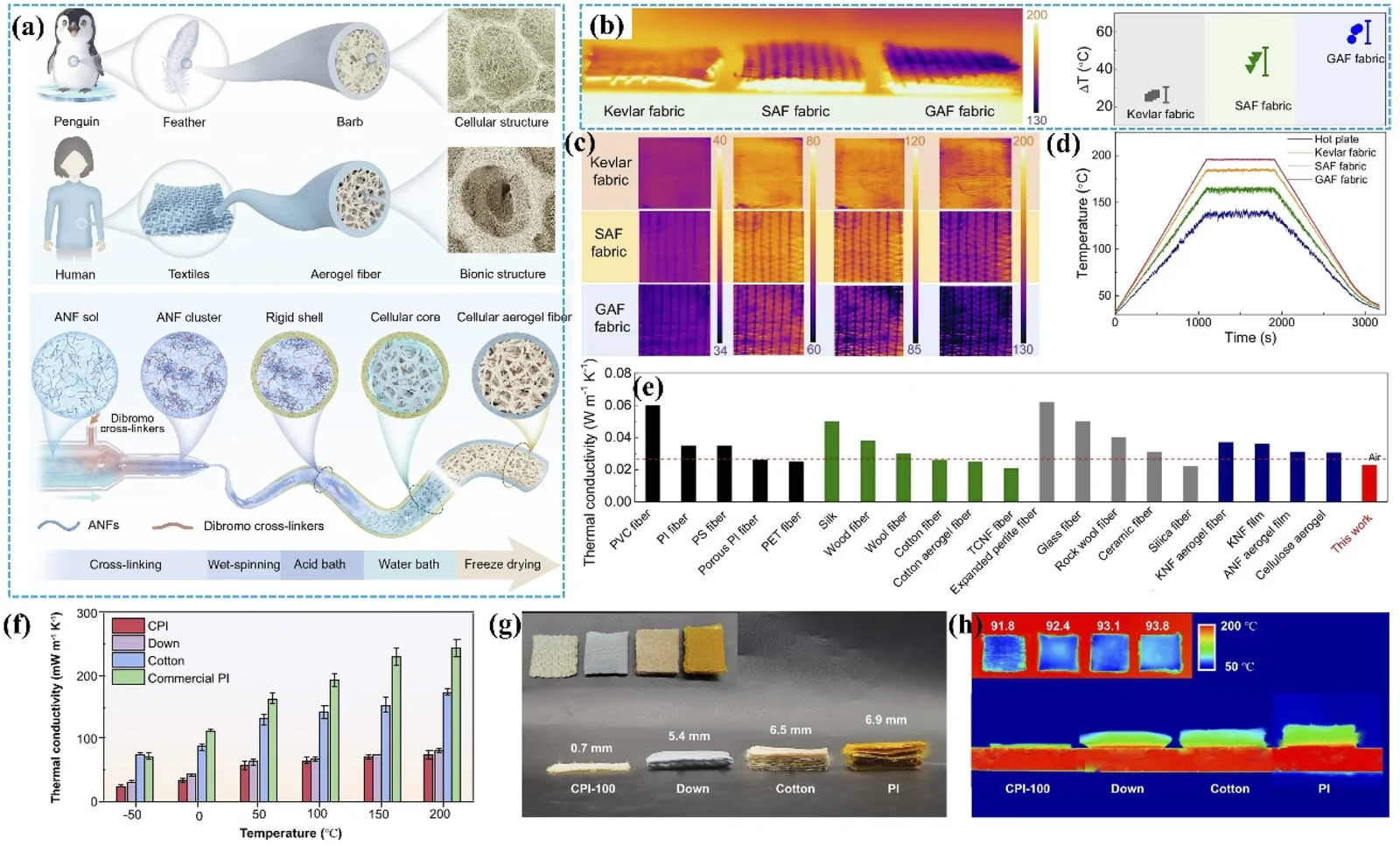
Radial structure–property relationships in wearable aerogel fibers and fabrics. (a) Penguin-feather-inspired cellular ANF fiber with a rigid shell and soft cellular core. (b–e) Infrared comparison of equal-mass and 0.5 mm-thick Kevlar, skin–core aerogel fiber (SAF), and gradient all-nanostructured aerogel fiber (GAF) fabrics; heating curves and conductivity comparison show the benefit of the gradient nanoporous interface. (f) Conductivity of CPI aerogel fabric, down, cotton, and commercial PI from −50 to 200 °C; (g and h) optical and infrared comparison of 0.7 mm CPI-100 fabric with 5.4 mm down, 6.5 mm cotton, and 6.9 mm PI on a 200 °C hot stage. (a) Reproduced with permission. Copyright 2026, Springer Nature.^165^ (b–e) Reproduced with permission. Copyright 2025, Springer Nature.^166^ (f–h) Reproduced with permission. Copyright 2023, Springer Nature.^163^

Fig. 11f–h provides a thickness-normalized textile comparison. A 0.7 mm CPI aerogel fabric produced a 108 °C temperature difference on a 200 °C hot stage, comparable to 5.4 mm down, 6.5 mm cotton, and 6.9 mm commercial PI. Its conductivity increased from 24.2 mW m^−1^ K^−1^ at −50 °C to 70.2 mW m^−1^ K^−1^ at 150 °C, whereas cotton increased from 75.2 to 153.2 mW m^−1^ K^−1^ and commercial PI from 82.4 to 230.1 mW m^−1^ K^−1^.^163^ The relevant advantage is thermal resistance at constrained thickness across temperature, not only the minimum room-temperature conductivity.

One-dimensional aerogel fibers are not the primary focus of this review, but they provide transferable design lessons. Core–shell, cellular, gradient, and coaxially encapsulated fibers place a robust sheath around a soft porous core, allowing knotting, weaving, and sewing while limiting radial heat flow.^165–174^ For three-dimensional aerogels, the same concept can be used as protective skins, graded edges, or fiber-rich attachment zones rather than as a dense matrix throughout the component.^44,99,175,176^

Active personal thermal management adds conductive heating or latent-heat storage to passive insulation.^9,53,177^ These functions should be spatially separated from the principal through-thickness barrier; otherwise they raise passive conductivity and create hot spots. Power density, temperature uniformity, burn safety, wash durability, and unpowered heat leakage must be reported together.^178,179^

### 5.3 Building insulation

Building insulation prioritizes low cost, large-area manufacture, fire and moisture safety, and service over decades. The plant-cell-wall-inspired cellulose composite is instructive because ambient drying limited shrinkage to 8.56% and retained a conductivity of 0.0381 W m^−1^ K^−1^,^23^ demonstrating that capillary stability can be more important than maximizing initial porosity. Waste cotton, office paper, starch/clay/cellulose, kapok/alginate, and rice-husk-derived systems broaden the feedstock base,^46–48,85,90,93^ but their value depends on controlling fiber-length distribution, ash content, moisture uptake, and batch variability.

Building performance is governed by assemblies rather than isolated coupons. Facings, joints, fasteners, mounting compression, and vapor barriers can bypass the pore network. Silica–cellulose and collagen/silica co-networks improve dimensional or moisture stability,^180–182^ whereas molecularly bridged glass-fiber/silica composites improve handling.^183^ These gains must be balanced against added density and interfacial solid conduction and verified behind realistic facings under repeated wet–dry cycles.^184^

Fire qualification further separates laboratory promise from building readiness. Mineral- or char-forming alginate, cellulose, and PVA networks can preserve a residue,^59–62^ but limiting oxygen index or a short torch image is insufficient. Cone calorimetry, smoke toxicity, flame spread, post-fire integrity, and wall-scale back-face temperature under simultaneous heat and moisture gradients are required.^185^

### 5.4 Transportation, batteries, and electronics

Transportation adds vibration, repeated compression, condensation, restricted thickness, and severe mass penalties. Industrial sea-island PPS ultrafine fibers assembled into corrugated aerogel felts provide a useful benchmark: the felt had a density of about 6.4 mg cm^−3^ and a conductivity near 28 mW m^−1^ K^−1^, tolerated more than 25% tensile strain, 95% compression, 180° bending, and over 10 000 cycles, and provided insulation comparable to down at roughly one-third the thickness.^20^ The conclusion is that scalable fibers become transport-ready only when the felt architecture retains thickness under combined bending and compression.

Battery and electronic protection requires a distinction between steady-state insulation and transient thermal-runaway management. Moisture-resistant PI membranes, shaped printed aerogels, and fluorinated PI/paraffin sandwiches address dielectric protection, geometric conformity, and latent-heat delay.^111,118,134^ A credible barrier must additionally be tested under localized heat flux, flame and vented gas, electrical potential, vibration, and module-level compression; room-temperature conductivity alone cannot predict the time to thermal propagation.

Conductive multifunctional aerogels require particular caution near electronics. PI/MXene, MXene/aramid/PI, ANF/CNT, and carbon-nanofiber/polyaniline networks add sensing or electromagnetic shielding,^9,105,131,153^ but their conductive paths should be segmented, encapsulated, or displaced from the protected surface. Dielectric isolation and passive conductivity must be re-evaluated after bending, vibration, humidity, and thermal cycling.

Fig. 12 exposes the central trade-off in ablative composites. Adding TiB_2_–B_4_C to a phenolic-aerogel/carbon-fiber skeleton increased room-temperature solid conduction (Fig. 12a and b), yet generated C–B_2_O_3_–TiO_2_–TiC protective phases during ablation. The optimized composite retained 58.8% mass at 1200 °C, preserved a residual strength of 3.44 MPa after static ablation, and reduced linear and mass ablation rates to 0.045 mm s^−1^ and 0.0115 g s^−1^, respectively (Fig. 12c and d).^186^ Carbonizing phenolic-fiber/RF aerogels from 1000 to 1600 °C increased bending strength from 1.92 to 3.62 MPa, but the high-temperature conductivity also rose; Fig. 12g shows an approximate range of 0.05–0.11 W m^−1^ K^−1^ at 400 °C and 0.17–0.29 W m^−1^ K^−1^ at 1800 °C.^187^ Thus, ceramic yield and carbon continuity improve ablation integrity but should be localized where the gain in hot-face survival justifies the solid-conduction penalty.

**Fig. 12:**
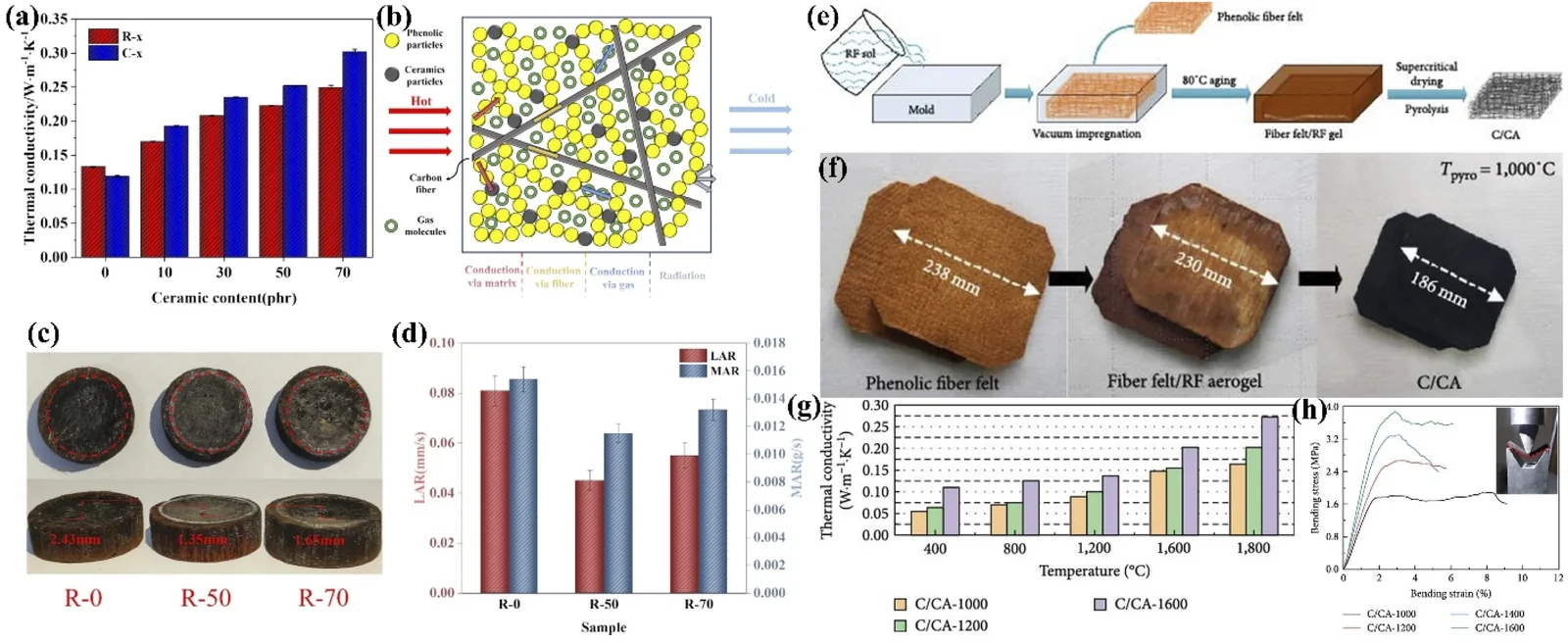
Strength-conduction trade-offs in ablative and carbonized fibrous aerogels. (a) Thermal conductivity before and after static ablation as ceramic content increases; (b) conductive, gaseous, and radiative pathways through the ceramizable phenolic composite; (c and d) post-ablation morphology and linear/mass ablation rates. (e and f) Vacuum impregnation, gelation, supercritical drying, and pyrolysis of phenolic-fiber/RF composites; (g) thermal conductivity from 400 to 1800 °C and (h) bending stress–strain curves as carbonization temperature increases from 1000 to 1600 °C. (a–d) Reproduced with permission. Copyright 2024, Springer Nature.^186^ (e–h) Reproduced with permission. Copyright 2023, Hindawi Limited.^187^

### 5.5 Aerospace and extreme environments

Extreme environments combine large thermal gradients, low pressure or vacuum, vibration, oxidation, and strict mass limits. Room-temperature conductivity is not sufficient because radiation grows rapidly with temperature and gas conduction changes with pressure. Polymer skeletons should be paired with a spatially limited inorganic hot face and evaluated by high-temperature/vacuum conductivity, back-face temperature, shrinkage, residue integrity, and recovery after coupled thermal–mechanical cycling.

PI provides broad-temperature resilience when curvature and crosslinking preserve the pore network. Curly PI nanofibers combined 24.5 mW m^−1^ K^−1^ conductivity with recovery after 1500 deformations,^18^ while gradient and double-phase PI/inorganic networks separate hot-face stability from a compliant core.^19,76,188,189^ The inorganic phase should remain below the connectivity required for a continuous heat path, except in the thin region intended to form a protective residue.^190–197^

Aramid and PBO complement PI through high specific strength and flame stability, but their practical benefit depends on regulated fibrillation and junction formation.^15,16,101^ At still higher temperatures, mullite-fiber-reinforced alumina–silica aerogels remained undeformed after exposure to 1500 °C and showed a thermal conductivity of 0.082 W m^−1^ K^−1^ at 1200 °C.^164^ This inorganic comparator defines the point at which a polymer-containing barrier must be designed for controlled transformation and residue integrity rather than retention of the original polymer.^198–200^

Deployable and space structures add folding and recovery requirements. Localized covalent fusion can preserve an ultralight network during repeated deployment,^14^ whereas gradient quartz-fiber and SiC-lattice sandwich systems separate a rigid hot face from a low-density core.^201,202^ Qualification should include vacuum conductivity, outgassing, radiation or atomic-oxygen stability where relevant, vibration, and repeated thermal-vacuum/deployment cycles.

### 5.6 Scalable manufacturing and sustainability

Scale-up changes the physics of freezing, solvent exchange, imidization, and drying because heat- and mass-transfer gradients can develop into macroscopic defects. The UV-enhanced dynamic gelation of crosslinked PI fibers illustrates how rapid structural fixation can mitigate this problem: hundreds of meters of fiber were produced within 7 h, compared with 49–94 h for earlier routes, while achieving a specific modulus of 390.9 kN m kg^−1^.^163^ Continuous textile-derived processing provides a complementary strategy. Industrial sea-island melt spinning produced uniform, high-aspect-ratio PPS fibers whose aerogel felts retained their performance over more than 10 000 cycles,^20^ while roll-to-roll twisting and knitting demonstrated the importance of tension control and protective surface layers during conversion.^203–205^ Nevertheless, rapid gelation or continuous production is meaningful only if the pore hierarchy and junction topology remain uniform during drying, cutting, sewing, lamination, and mounting.

Scalability must also be evaluated together with environmental impact. Renewable or waste-derived feedstocks alone do not guarantee sustainability because freeze-drying energy, solvent toxicity and recovery, wash-water consumption, inorganic additives, production yield, and end-of-life separation may offset their benefits. Ambient-pressure drying, solvent recovery, low-solids forming, and recyclable PI networks provide potentially transferable solutions across polymer classes.^23,24^ Waste cotton, office paper, rice-husk/plastic waste, and tire-derived fibers further demonstrate the feasibility of circular feedstocks,^46,48,93,109,206^ although contamination levels, material recovery yields, and cradle-to-gate energy consumption must be quantified before environmental advantages can be established.

Table 3 consolidates application-specific evidence rather than ranking materials solely by room-temperature thermal conductivity. Representative examples include thickness-normalized clothing insulation,^163^ a PPS felt with a density of 6.4 mg cm^−3^ that combines a thermal conductivity of approximately 28 mW m^−1^ K^−1^ with durability over more than 10 000 cycles,^20^ PI electronic barriers integrating thermal insulation with dielectric or latent-heat functions,^111,118^ and a mullite composite retaining a thermal conductivity of 0.082 W m^−1^ K^−1^ at 1200 °C.^164^ Across these applications, the central translational gap is not the lack of low-conductivity formulations, but the limited evidence that their insulation performance survives coupled thermal, mechanical, and environmental loading. The available results support a common design principle: continuous solid heat-transfer pathways should be minimized, while localized reinforcement should be introduced where necessary to preserve pore geometry, suppress radiative heat transfer, or generate a protective residue. Engineering adoption requires material selection based on retained thermal resistance under application-specific conditions, together with defined operating envelopes, assembly-scale validation, statistically controlled manufacturing, and transparent lifecycle assessment.

**Table 3:** Application-specific quantitative benchmarks, design priorities, and validation requirements for polymer fibrous aerogels and relevant high-temperature hybrids

Application	Representative system and source	Quantitative benchmark	Design implication and dominant constraints	Essential application-level validation	Ref.
Clothing and personal thermal management	Cellular ANF aerogel fiber; gradient all-nanostructured aramid fiber; crosslinked PI (CPI) aerogel fabric	ANF fiber: 74.6 MPa tensile strength; ending/compression stresses of 33.8/39.8 kPa; conductivity 28.4–32.1 mW m^−1^ K^−1^, increasing only from 28.4 to 29.7 mW m^−1^ K^−1^ between 30% and 90% RH. A 0.7 mm CPI fabric produced a 108 °C temperature difference on a 200 °C stage and showed 24.2–70.2 mW m^−1^ K^−1^ from −50 to 150°	Radial cellular or gradient structures reconcile tensile continuity with soft bending and low radial heat flow. The principal constraints are thickness, areal mass, breathability, sweat, washing, abrasion, and comfort	Areal thermal resistance at fixed thickness and compression; water-vapor transport; wash/abrasion durability; bending and sewing fatigue; skin-side temperature; passive leakage when active functions are unpowered	168, 170 and 171
Building envelopes	Ambient-dried, plant-cell-wall-inspired cellulose composite aerogel	Drying shrinkage was limited to 8.56%, while thermal conductivity remained 0.0381 W m^−1^ K^−1^	Collapse resistance and low-cost large-area drying can be more valuable than a lower coupon-scale conductivity. Fire/smoke safety, humidity, creep, facings, joints, and multidecade stability dominate selection	Assembly U-value or heat flow with facings, joints, and fasteners; wet–dry and freeze–thaw aging; creep under mounting pressure; cone calorimetry, smoke toxicity, flame spread; batch-to-batch variability	23
Transportation	Industrial sea-island melt-spun PPS ultrafine-fiber corrugated aerogel felt	Density approximately 6.4 mg cm^−3^; conductivity approximately 28 mW m^−1^ K^−1^; more than 25% tensile strain, 95% compression, 180° bending, and over 10 000 cycles; insulation comparable to down at approximately one-third the thickness	A resilient felt must retain thickness while accommodating bending, vibration, condensation, and attachment. Scalable fiber manufacture alone is insufficient if corrugation or node geometry collapses during service	Conductivity and thickness after combined vibration/compression aging; condensation and temperature cycling; attachment and seam effects; smoke and flame response; impact retention; thermal–acoustic performance	20
Batteries and electronics	Moisture-resistant PI aerogel membrane; fluorinated PI aerogel/paraffin sandwich; 3D-printed nanocellulose/aramid aerogel	PI membrane: conductivity 35.4 mW m^−1^ K^−1^ and dielectric constant 1.32. Sandwich composite: dielectric constant 1.04 in the directional lower layer and paraffin latent heat of 242.7 J g^−1^ in the upper layer. Shapeable printed hybrid: conductivity 0.032 W m^−1^ K^−1^	Steady insulation, electrical isolation, geometric conformity, and transient heat storage should be spatially separated. Confined volume, localized heat flux, vented gas, flame, leakage, and conductive additives create coupled failure modes	Module-scale propagation delay and back-face temperature; localized heat-flux/flame/vented-gas exposure; dielectric strength and leakage current; PCM leakage and cycling; vibration and compression under electrical bias	116, 123 and 139
Aerospace and extreme thermal protection	Mullite-fiber-reinforced alumina–silica aerogel; TiB_2_–B_4_C-modified phenolic-aerogel/carbon-fiber composite	The mullite composite remained undeformed after 1500 °C exposure and showed 0.082 W m^−1^ K^−1^ at 1200 °C. The ceramizable phenolic composite retained 58.8% mass at 1200 °C, 3.44 MPa residual strength, and linear/mass ablation rates of 0.045 mm s^−1^/0.0115 g s^−1^	A localized inorganic or ceramizable hot face can preserve residue integrity even when it raises room-temperature solid conduction. Radiation, vacuum, oxidation, ablation, vibration, and strict mass limits govern the architecture	High-temperature/vacuum conductivity; transient back-face temperature and heat flux; shrinkage and residue integrity; ablation rate; outgassing; vibration; thermal-vacuum cycling; atomic-oxygen/radiation durability where applicable	169 and 191
Scalable manufacturing and circular deployment	UV-enhanced dynamic gelation of CPI aerogel fibers; industrial PPS sea-island fiber/felt route; ambient-dried or recyclable networks	Hundreds of meters of CPI fiber were produced within 7 h, compared with 49–94 h for earlier routes, with a specific modulus of 390.9 kN m kg^−1^. Industrial PPS felts retained performance through more than 10 000 cycles	Scale-up must fix the pore structure before handling and maintain it through cutting, sewing, lamination, and mounting. Drying energy, solvent recovery, yield, feedstock variability, and end-of-life separation determine sustainability	Roll-to-roll distributions of diameter, density, thickness, pore size, and conductivity; process yield and defect mapping; solvent/water recovery; energy per unit area; lifecycle inventory; repair, separation, and recycling yield	20, 23, 24 and 168

## 6 Conclusions and perspectives

### 6.1 From polymer chemistry to a structural design window

Polymer fibrous aerogels do not derive their performance from low density or polymer identity alone. The evidence reviewed here shows a continuous causal chain: molecular structure defines fiber formation and interfacial chemistry; processing establishes dispersion, orientation, junctions, and pore connectivity; these features redistribute solid, gaseous, radiative, convective, and moisture-mediated heat transfer; and service exposure determines whether the architecture is retained. Cellulose offers aqueous renewable processing but requires moisture and fire control; aramid and PBO provide persistent high-temperature skeletons but impose demanding fibrillation; PI and PAI allow broad molecular and topological design but require control of imidization and shrinkage; and other thermoplastics or thermosets expand manufacturing and fire-response options within narrower operating windows.

Across these polymer families, the most transferable conclusion is the existence of a structural design window. Sparse high-aspect-ratio fibers, controlled curvature, hierarchical but nonpercolating macropores, localized strong junctions, and spatially confined functional phases can preserve a low-conductivity gas-filled network. Overrefinement, excessive alignment, continuous crosslinking, or filler percolation shifts the same structure toward aggregation, brittleness, pore loss, or thermal bridging. The design objective is not to maximize porosity, bond strength, or additive loading independently, but to minimize thermally effective connectivity while retaining mechanically redundant load paths.

### 6.2 Establishing causal processing–Structure–transport relations

The first unresolved problem is the weak causal connection between process variables and retained thermal performance. Future studies should quantify how fiber-size distributions, bundle fraction, junction coordination and area, pore-throat connectivity, anisotropy, shrinkage, and hybrid-phase topology evolve from the wet state through drying and service. These descriptors should then be related separately to solid conduction, confined-gas transport, radiation, permeability, and moisture sorption. Direction-, pressure-, temperature-, and humidity-dependent conductivity should replace isolated room-temperature values. Mechanistic models and data-driven methods will be useful only when datasets report processing histories, uncertainty, negative results, aging, and structural evolution rather than nominal composition and record values alone.

### 6.3 Balancing insulation, mechanics, fire safety, and moisture resistance

The second unresolved problem is the frequent improvement of one function at the expense of another. Junctions, coatings, opacifiers, and flame retardants can preserve dimensions, block radiation, or promote char, yet they may also increase solid conduction, restrict fiber motion, or impede recyclability. Future work should optimize spatial distribution instead of bulk loading and evaluate retained thickness and conductivity after cyclic compression, humidity, thermal aging, wet–dry or freeze–thaw cycling, and realistic heat-flux or fire exposure. Back-face temperature, smoke release, residue integrity, coating retention, and post-exposure mechanics should accompany conventional conductivity and flame metrics. The decisive performance measure is the thermal resistance that remains after coupled service damage.

### 6.4 Scalable manufacturing and application qualification

The third unresolved problem is the gap between laboratory specimens and qualified components. Scale-up must control heat and mass transfer through thick bodies, solvent recovery, imidization, dimensional tolerance, and handling from wet forming to installation. Continuous fiber production, roll-to-roll assembly, direct ink writing, ambient-pressure drying, localized welding, and recyclable networks provide promising routes, but they require batch statistics, energy and solvent balances, and component-level quality control. Application validation must include seams and sewing zones in clothing, joints and fasteners in buildings, mounting pressure and vibration in transportation, transient heat flux and dielectric safety in batteries, and vacuum or thermal-vacuum cycling in aerospace. Addressing these linked manufacturing and qualification requirements will determine whether polymer fibrous aerogels become reliable insulation systems rather than isolated high-performance specimens.

## Author contributions

Xuepeng Ni: writing – original draft, investigation, formal analysis, data curation, conceptualization. Jingyu Guan: investigation, data curation. Jie Dou and Xiaojuan Wang: visualization, investigation, data curation. Liyin Hou and Jiaming Guo: investigation, formal analysis, data curation. Qi Wang and Caixia Ren: writing – review & editing, project administration, data curation, conceptualization, visualization, investigation. Zhe Cui: writing – review & editing, supervision, resources, conceptualization.

## Conflicts of interest

The authors declare that they have no known competing financial interests or personal relationships that could have appeared to influence the work reported in this paper.

## Data Availability

No primary research results, software or code have been included and no new data were generated or analysed as part of this review.
